# Designing privacy-friendly digital whiteboards for mediation of clinical progress

**DOI:** 10.1186/1472-6947-14-27

**Published:** 2014-04-04

**Authors:** Erlend Andreas Gjære, Børge Lillebo

**Affiliations:** 1 SINTEF ICT, Department of Software Engineering, Safety and Security, Po. box 4760 Sluppen, N-7465 Trondheim, Norway; 2 Norwegian EPR Research Centre, Department of Neuroscience, Faculty of Medicine, Norwegian University of Science and Technology, Trondheim, Norway

## Abstract

**Background:**

In hospitals, digital versions of dry-erase whiteboards are increasingly becoming more common. One of the purposes with such whiteboards is to support coordination of care by augmenting visibility and availability of clinical information. However, clinical information usually concerns patients and is regarded as sensitive personal health information, meaning that it should be access controlled. The purpose of this study is to explore how digital whiteboards can be designed for supporting coordination of care, by providing clinicians with useful information in a usable way, and at the same time protect patient privacy.

**Methods:**

A demo application was designed, demonstrated and evaluated iteratively. In total, 15 professional ward nurses role-played a scenario in which the application played a central part. Afterwards, the participants were interviewed. All interviews were recorded, transcribed verbatim, and analysed qualitatively.

**Results:**

The participants valued having updated clinical information presented on a digital whiteboard, even if the information was de-identified and abstracted. According to the participants, such information could possibly improve inter-departmental communication, reduce the number of electronic health record-logins, and make nurses more rapidly aware of new information. The participants expected that they would be able to re-identify much of the de-identified information in real situations based on their insight into their patients’ recent and expected care activities. Moreover, they also valued being able to easily access more detailed information and verify patient identities. While abstraction and de-identification was regarded to sufficiently protect the patients’ privacy, the nurses also pointed out the importance of having control over what can be seen by other patients and passers-by if detailed medical information was accessed on a digital whiteboard.

**Conclusions:**

Presenting updated information from patient care activities on a digital whiteboard in a de-identified and abstracted format may support coordination of care at a hospital ward without compromising patient privacy.

## Background

Many advances in health care can be attributed to the rise in specialisation. However, this fragmentation, in a sense where more people and professions are required for the treatment of every single patient, has not been without side-effects [1]. Hospitals have become complex institutions with challenging coordination needs [2]. According to Bates and Gawande, “the fundamental difficulty of modern medical care is execution. Providing reliable, efficient, individualized care requires a degree of mastery of data and coordination that will be achievable only with the increased use of information technology” [3] (p. 2533).

Organisation theory states that difficult coordination is best met by enabling those involved to stay aware of each other in order to continuously adapt to each other’s work [4]. This kind of awareness of “what is happening around you and understanding what that information means to you now and in the future” has been referred to as situation awareness [5] (p. 13). Studies in various domains have demonstrated that technology can improve situation awareness and lead to better performance [6]. Within hospitals, staying aware of what is going on beyond a person’s immediate workspace has been found to be an important factor for ensuring smooth perioperative coordination [7]. The use of digital whiteboards has been suggested as a means of improving awareness and reducing the effort of clinicians to monitor clinical work [8]. While traditional dry-erase whiteboards are widely used in hospital settings [9], digital whiteboards are less common [10-20]. However, compared to traditional whiteboards, digital whiteboards offer some compelling features such as real-time display of patient-centred information, rapid access to more detailed information, and enhanced visibility and availability of clinical information at multiple locations [15]. This, on the other hand, requires whiteboards to somehow mitigate threats to the confidentiality of personal health information (PHI) [21,22], including information that concerns patient care activities.

Manual encoding of information that needs special protection seems to be a feature that has been lost in the transition from analogue to digital whiteboards. Such protection could for instance be expressing some particular condition with an arbitrary-looking red dot, that only individuals in the specific care-providing team know the meaning of [22]. Instead, the digital whiteboard’s contents would normally be protected using computational access control mechanisms, which can indeed be much more secure. However, by requiring authentication before any disclosure, users are prevented from simply obtaining a quick overview when passing by or looking from a distance. Conventional authentication techniques may also take time and attention away from the user’s original task, and do not fit well into the nature of clinical work [23].

Rather than relying on traditional authentication techniques alone, or even bypassing the entire patient privacy issue by only having shared digital whiteboards in restricted areas [20,24], we wanted to investigate how de-identified and abstracted pieces of information can be used to attract timely attention from its relevant recipients. By abstraction we mean that the clinician is informed about the occurrence of a clinical activity (e.g. “Imaging results are available”) rather than the specific content of that activity (e.g. “The CT-scan reveals necrotic pancreatitis”). De-identification is usually associated with the scrubbing process applied to medical data before such data can be disclosed from a health care organisation to an entity not being directly involved in patient care, e.g. researchers. This involves the removal of patient identifying information from the data set, e.g. by removing names and birth dates, or by removing entire entries that are unique beyond a certain threshold (k-anonymity) [25]. De-identification hence intends to balance utility value and protection of information. A concept of *flexible* de-identification has been proposed as a possible solution to patient privacy issues when information is shared in real-time between collaborating health care personnel [26]. The flexibility lies in that whenever any de-identified piece of information is found to be relevant, more information–including explicit patient identifiers–can be accessed through secure authentication mechanisms. An analogy for this is the lock screen on a smart-phone, displaying icons to notify the user about a new message and possibly its sender, and then requiring a PIN-code for accessing the full message. The question of information relevance, however, becomes more difficult when the screen is shared among groups and the recipients are not known to the system. In addition, the sensitive nature of the information effectively halts any kind of open broadcasting that could give patients doubts about sharing their personal stories with their health care providers.

In some early interviews [27], we found that clinicians who discuss a patient and do not want to be overheard, initially may use (combinations of) non-explicit identifiers for the patient, such as medical problem, age group and gender. Treatment history, the current situation and expected future care activities, could also be utilised when approaching precise identification during a conversation, e.g. ‘he who had a gastric bypass yesterday’. Likewise, we hypothesised that digital whiteboards that display de-identified information may be useful if the patients are known to the clinician–while not being meaningful to others. The objective of this study was to: 1) Understand what information supports surgical ward nurses in managing perioperative care; and: 2) Explore how–and how much of–such information can be presented on a digital whiteboard, without compromising the privacy of the patients.

## Methods

In this study we adhered to the Design science research process (DSRP) model [28]. We conducted three iterations of design/redesign, demonstration and evaluation. Our main design artefact was a digital whiteboard that displayed an overview of patient care events pertaining to eight patients at a surgical ward unit. The digital whiteboard was intended to hang in a non-restricted area where the ward nurses easily would see it, and provide the nurses with sufficient information to support coordination of their nursing activities. On this basis, the digital whiteboard would also be exposed to unintended users such as patients and passers-by, requiring special attention to what information is disclosed and how it is presented.

A preliminary risk-based evaluation of access control approaches for groups [29] provided us with a privacy-oriented foundation for exploring the whiteboard designs. Based on a simple approach where the whiteboard’s physical location would determine the default privacy level, the recommendation was given to extend this with support for either handheld devices or small, on-screen pop-up windows as means to access sensitive information in a more private manner. The digital whiteboard was accordingly designed to support perception of any new information's availability, but by default all openly accessible information was de-identified and abstracted to a level of detail which could be allowed in non-restricted areas at any time. This was a fundamental design feature of the digital whiteboard throughout our study, with varying levels of abstraction and de-identification demonstrated and evaluated throughout the iterations (Table 1). To identify a minimum level of information detail needed by default on the whiteboard for it to be useful, we isolated the location-based approach in the first iteration. To give access to more detailed medical information and disclosing the patient identity related to each care event, a desktop computer was provided here just like the nurses were already used to having one at the ward. A mobile phone was then introduced as a handheld device extension (second iteration). Integrated interactive functionality implemented pop-ups as protection from prying eyes (third iteration). Our study did not aim at comparing these auxiliary technologies in order to come up with a winner. This variety was rather a means to broaden the combined feedback from the participants, still focussing primarily on the digital whiteboard and how to balance visibility of clinical information against patient privacy.

**Table 1:** Comparison of the three iterations in our study

	**First iteration**	**Second iteration**	**Third iteration**
**Main artefact**	Digital whiteboard	Digital whiteboard	Digital whiteboard
**Prototype runtime**	PowerPoint	Web browser	Web browser
**Event organisation**	Combined feed (8 patients)	Combined feed (8 patients)	8 individual feeds
**Supplementary artefact**	Desktop computer (access patient identities and more detailed event information)	Personal mobile phone (receive more detailed event information)	None
**Authentication**	ID-card + username/password (both on desktop computer)	ID-card (on digital whiteboard) + personal mobile phone	ID-card + PIN-code (both on digital whiteboard)
**Participants**	4	3	8

### The artefacts

The first artefact consisted of only static graphics. It was developed with graphical software (Inkscape), and demonstrated as a series of images (using Microsoft PowerPoint) that revealed new information step by step in correspondence with a clinical scenario. The second and third artefacts were developed using HTML, CSS and JavaScript, and demonstrated using a standard web browser (Google Chrome). The third iteration artefact is provided in Additional file 1.

**Figure 1:**
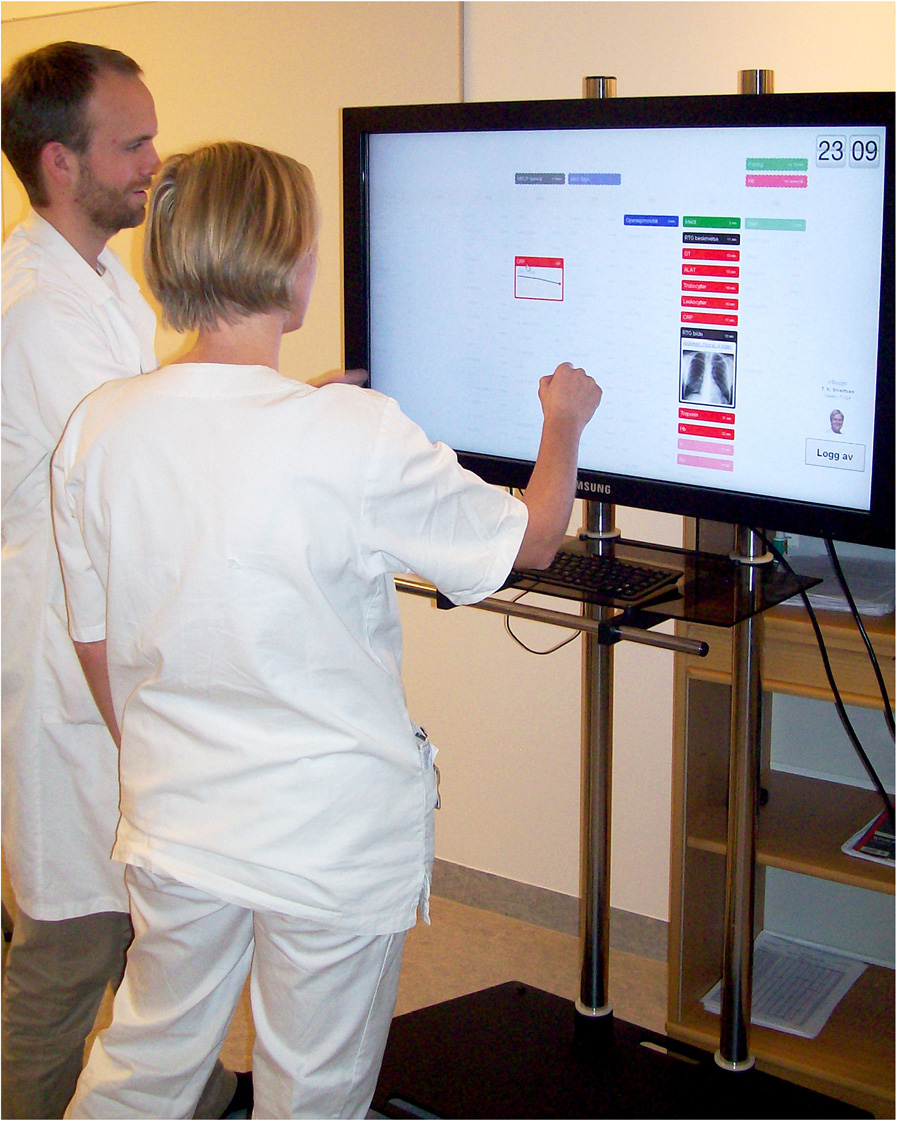
**Digital whiteboard.** Participant interacting with a digital whiteboard during a demonstration session, accompanied by one of the authors.

Demonstrations were primarily mediated through a 40" touch-enabled screen with HD resolution (1080p), infrared-based touch technology and 178° viewing angle. The screen was mounted on a foot stand with wheels, approximately 140 cm from the ground, as shown in Figure 1.

The information conveyed by the artefacts was abstractly differentiated through five event categories, each presented with a corresponding icon and colour. Lab events (red) were represented with a blood drop icon, indicating availability of a new blood test/lab result. Imaging events (black) used a small radiologic image, indicating progress updates for X-Ray, CT, MRI or ultrasound investigations. Medical record events (blue) had a paper sheet icon, and could arise whenever something was recorded in a patient’s electronic health record (EHR). Operation events (green) displayed a knife icon, stating real-time progress at certain pre-, per- and post-operative milestones. We also added ‘comment events’ (yellow) which were intended to resemble post-it notes, i.e. short messages between collaborating clinicians regarding events or activities that would not be included in the other event categories.

### First iteration: overview on a digital whiteboard, details on a desktop computer

**Figure 2:**
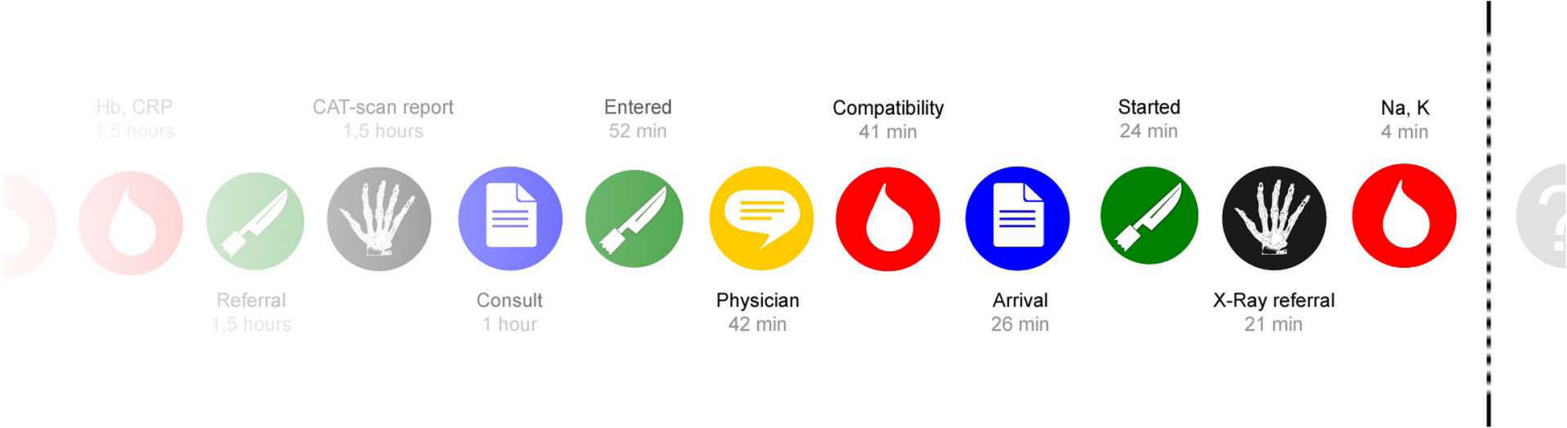
**First iteration, digital whiteboard.** This multi-patient, de-identified trajectory of care activities was displayed on a digital whiteboard located in the simulated ward corridor.

In our first iteration we visualised a multi-patient, horizontal trajectory of de-identified and abstracted recent care events on the digital whiteboard (Figure 2).

**Figure 3:**
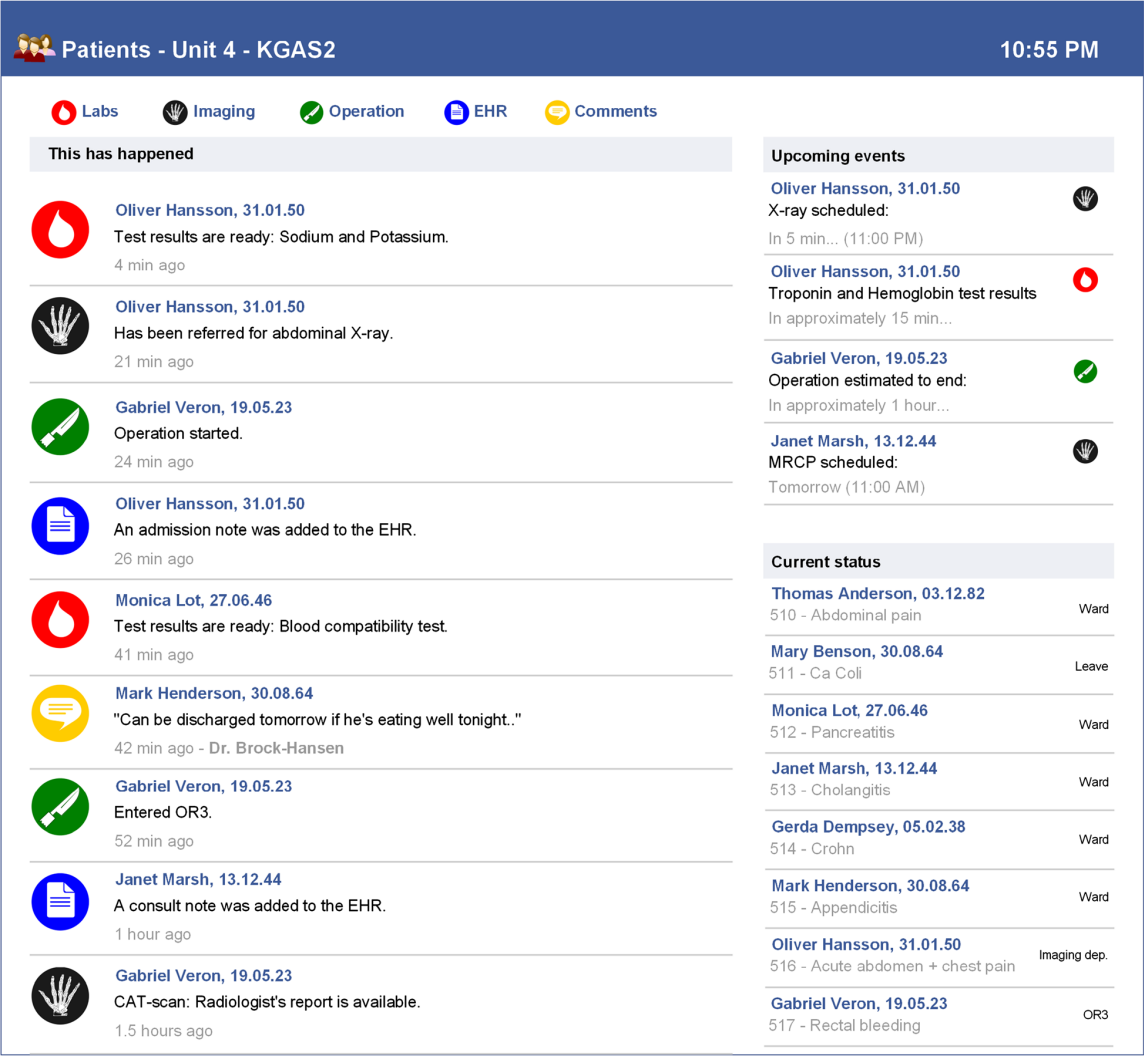
**First iteration, desktop view.** The desktop computer view of the prototyped application.

In addition, a desktop computer (PC) with a multi-patient, vertical trajectory of the same events was available at the central desk (Figure 3). The PC provided the nurse with medical details and patient identity corresponding to the de-identified and abstracted events that were shown on the digital whiteboard. The PC also presented a list of expected future events including an estimated event onset time. While the digital whiteboard was located in the corridor for high visibility purposes, the PC was protected with ID-card and password-based authentication, located at the central desk were nurses typically would have access to other clinical information systems (e.g. EHR with laboratory modules, radiology information system, operating room scheduling system). Actual access to these systems was not integrated in our demonstration sessions, and therefore any information that participants would normally find here (and could not find in our artefacts) was provided orally by the facilitator upon request.

### Second iteration: overview on a digital whiteboard, details on a mobile phone

**Figure 4:**
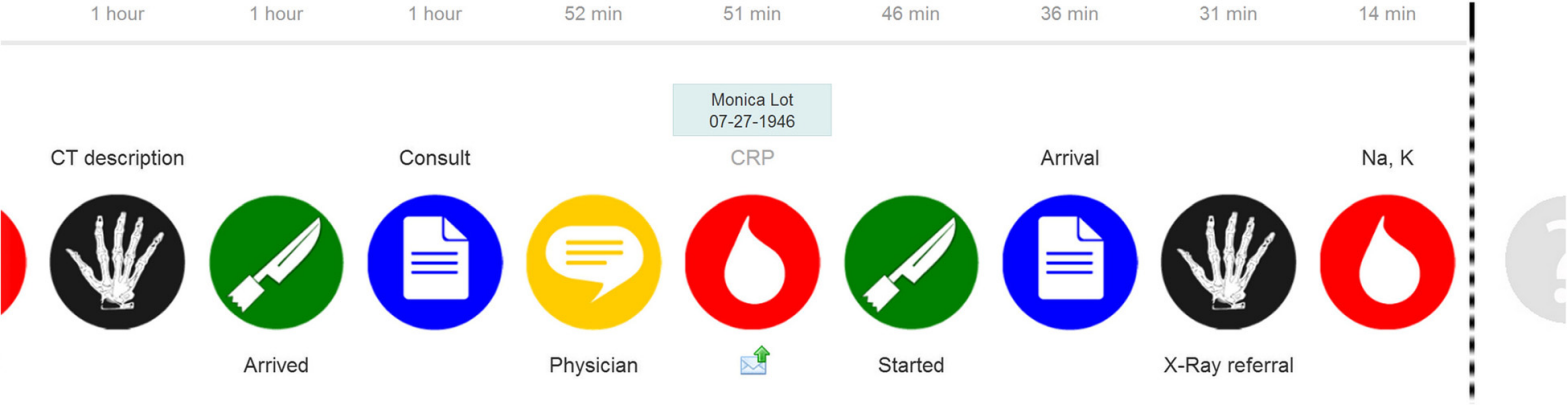
**Second iteration, digital whiteboard.** By swiping their ID-card and touching an event icon, the participants could identify the patient and request more detailed information to be sent to their mobile phone.

**Figure 5:**
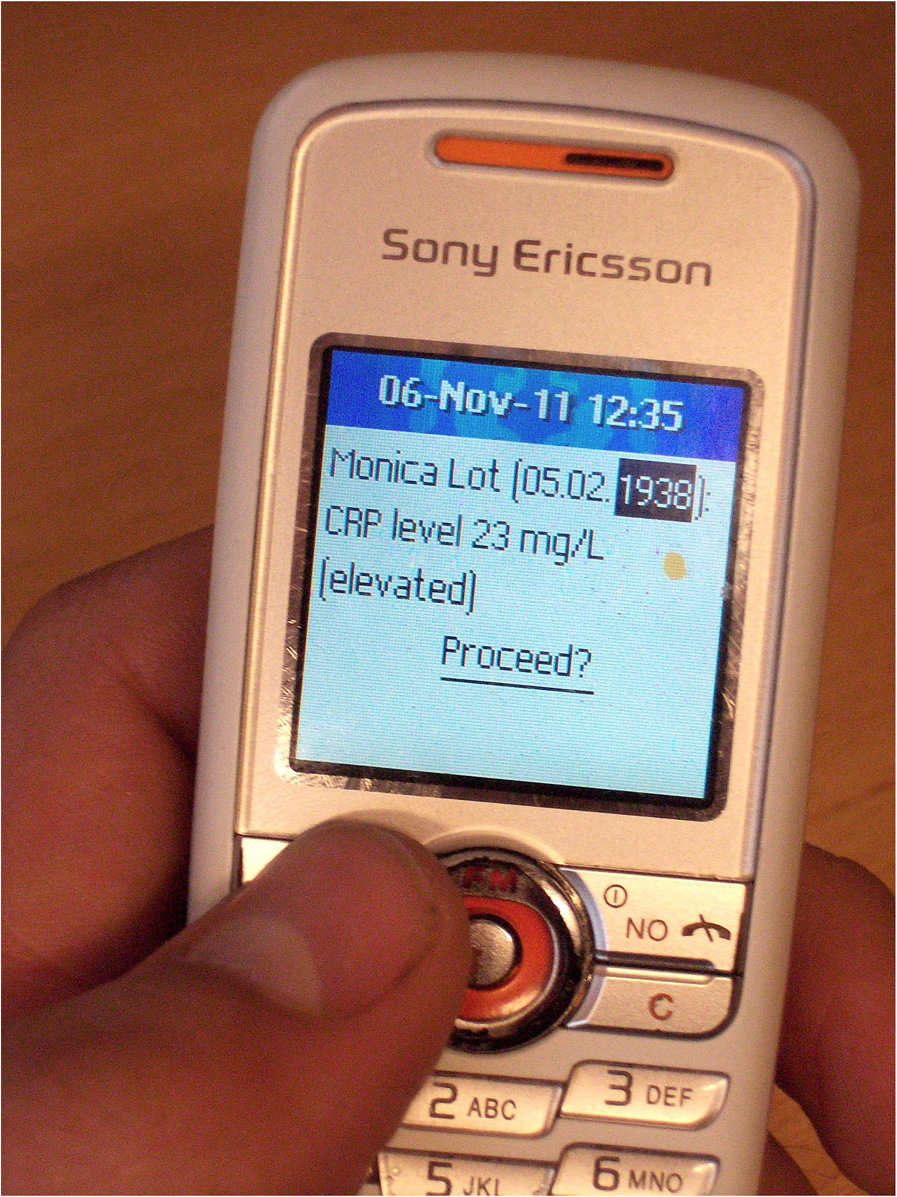
**Second iteration, mobile phone.** Example of message received on mobile phone, disclosing more detailed event information.

The digital whiteboard now offered some interactive functionality. Participants could identify the patient by swiping their personal identification card and touching events on the digital whiteboard, after which the name of the patient would appear (Figure 4, notice the fifth event from the right). The central desktop computer from the first iteration was replaced with a personal mobile phone. Thus, to disclose detailed event information the nurse could touch an event on the digital whiteboard and drag the finger to an emerging envelope below it (Figure 4). The medical details associated with that event would then appear on the mobile phone (Figure 5).

### Third iteration: both overview and details on a digital whiteboard

**Figure 6:**
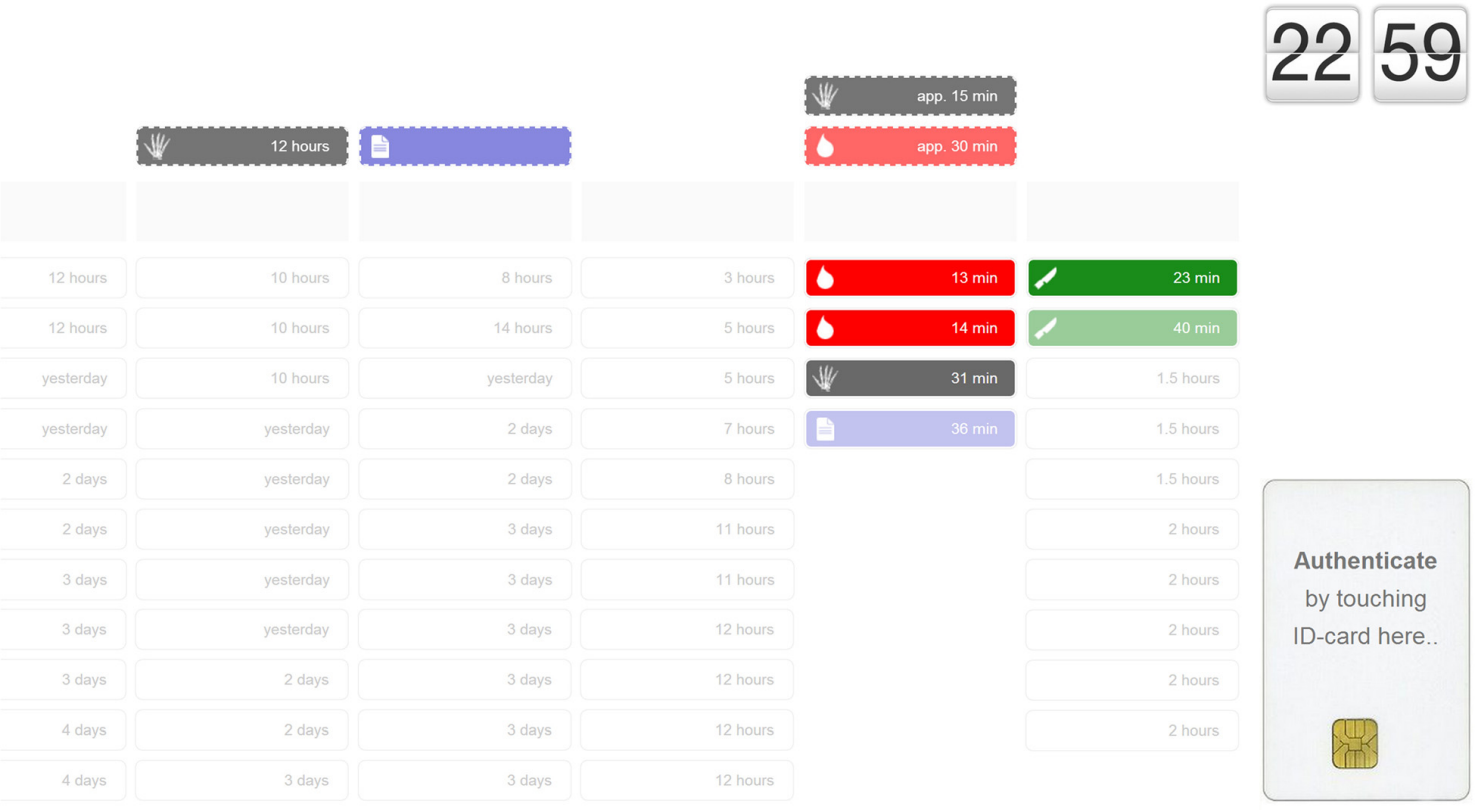
**Third iteration.** Vertical layout with one column for each patient trajectory, de-identified by default. Expected future events are located above the horizontal divider, with outlines dashed.

In the third iteration the digital whiteboard offered even more interactivity, and no other supplementary artefacts were available. Events were presented in eight vertical, single-patient trajectories. Thus, each patient at the unit had a designated column where new information would be presented (Figure 6). The most recent care events were located above older events, and expected future events were located at the very top of each column. Past and future events were separated by a grey placeholder row that contained more explicit identifiers such as room number or patient name. Still, neither of these identifiers were presented on the digital whiteboard by default. Only event categories (e.g. lab event, imaging event) and time-stamps were displayed by default–openly accessible to anyone passing by. A corresponding colour and icon were shown only for events that had occurred during the last hour (older information was grey), to support perceptual discrimination of old and new events.

**Figure 7:**
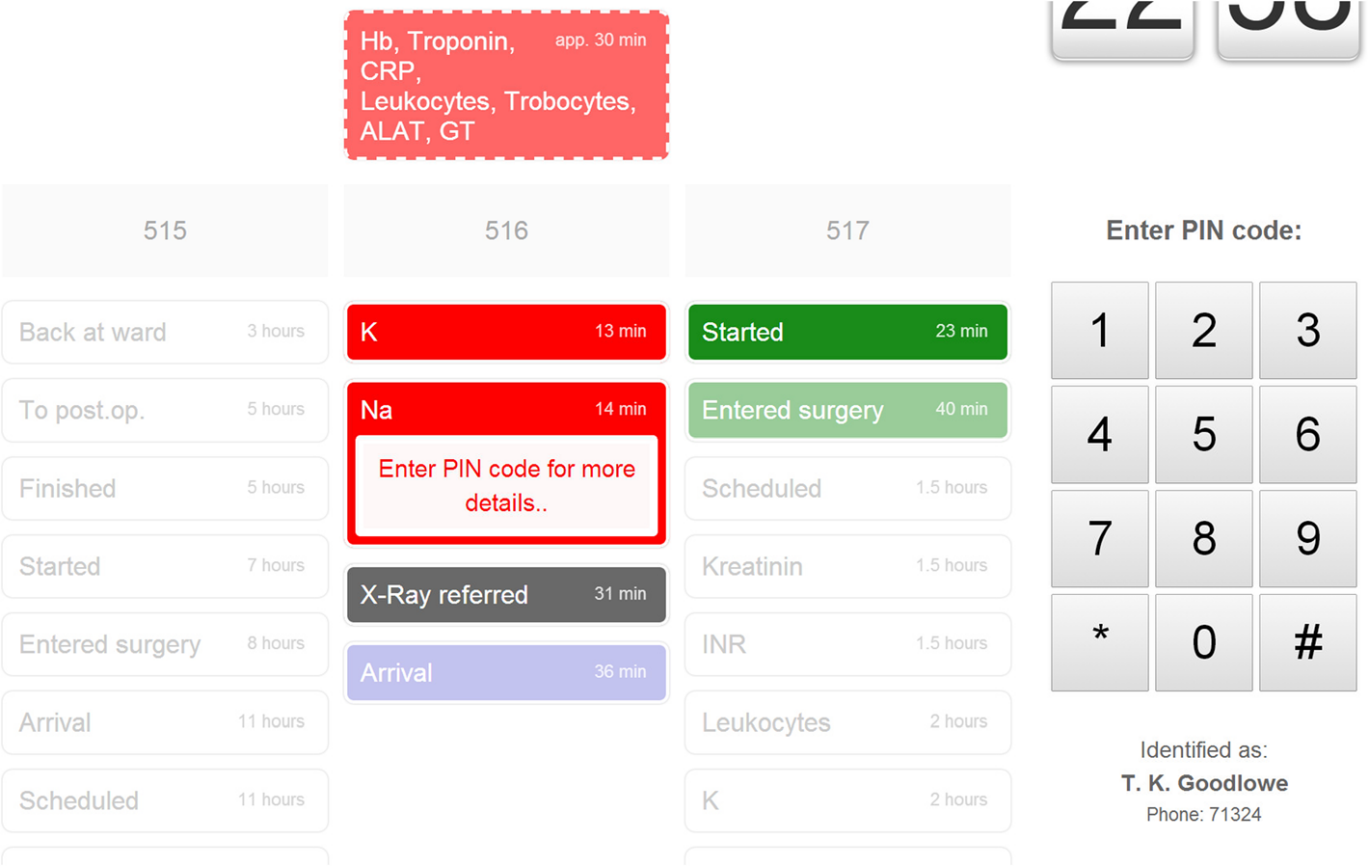
**More details on digital whiteboard.** Authentication with ID-card disclosed more detailed event information, but explicit identifiers and related medical details were hidden until the PIN-code had been entered.

By swiping their ID-card, participants could see room numbers and a specification of the event (e.g. below room number 516 in Figure 7–a lab event for sodium results). To access patient identities and detailed medical information, participants had to enter their PIN-code via an on-screen panel appearing to the right. Patient names would subsequently appear if the participant touched the room number label, and disappear when untouched. Moreover, when tapping a particular event, the ‘event-box’ would expand to disclose detailed medical information (e.g. “Sodium 140 mmol/L”). For lab results we also included a trend graph if there were more than one result for that particular analysis. Imaging events would open the associated image(s) or report on the digital whiteboard, while the medical record events would disclose the complete record note. Operation events could similarly disclose a note on the planned or performed operation. Our rationale for adding this richness of medical details was partly to explore the boundaries between a coordination support system and other clinical information systems, and partly to augment focus on the privacy aspect of accessing detailed clinical information on a digital whiteboard in a non-restricted area.

### Demonstration

#### Participants

All participants in the study were nurses at an 800-bed Norwegian university hospital. Participant sampling was purposive rather than representative. Each participant had at least one year of experience from ward work, and were recruited from either a gastro-surgical ward (‘standard ward’) or an observational ward (‘high throughput ward’). This sampling strategy ensured that the participants were familiar with coordination of care, albeit with some variety in work practices.

The observational ward employs a high-flow policy, admitting only patients expected to be discharged within 24 hours. Organisationally, these patients belong to multiple departments, including the gastro-surgical. At both of these types of wards, two or three nurses usually collaborate in units that comprise eight patients. These nurses share working environment and assist each other whenever needed. The nurses have frequent contact with the patients, and they often communicate with other departments to coordinate care. Whenever important changes in patient status occur or new test results are available, the nurses notify the responsible physician who is usually not co-located with the patients.

Participation was voluntary and was carried out during working hours in agreement with managerial staff. None of the nurses participated in more than one iteration throughout our study.

#### Procedure

Demonstrations took place in a setting configured to resemble a surgical ward unit, including a corridor, a central desk and patient rooms. Each demonstration session started by simulating a realistic handover meeting at the beginning of a night shift. The participant played the role of an incoming nurse, and one of the investigators (BL) played the outgoing nurse. First, the outgoing nurse informed the incoming nurse about the patients at the ward. This included a summary of present illness, what had been done, what needed further attention and which care activities awaited for each of the patients. There were no actual patients in the experiment, but a total of eight fictive patients with realistic surgical problems were presented orally during the handover. A printed list with room numbers, tentative diagnoses, and treatment plans for each of the eight patients supplemented the oral presentation (Table 2). This list had an authentic layout compared to the lists in use at the hospital from which the participants were recruited. The participants were further encouraged to take notes, ask questions and do whatever they were used to from actual handover meetings. The demonstration proceeded with a simulation of ward work during which the participant was to actively use the artefact to familiarise with its functionality. A pre-defined sequence of information updates were made available through the artefact interspersed with visiting patient rooms (Table 3). During patient visits the nurse was brought to another room–out of sight of the artefacts. Instead of meeting real patients there, the nurse was told that time advanced 5 or 10 minutes while working inside that room, e.g. carrying out a urine catheterisation. Every time the nurse returned to the corridor, the clock on the digital whiteboard would reflect the time that should have passed, and new information would have been added (with one exception). Hence, the nurse had to discover any new information and possibly access more detailed information. Whenever participants explained that they normally would take certain actions based on that new information, e.g. inform patient or responsible physician about new test results, they were told that they had now carried out that action (without actually doing it).

**Table 2:** List of patients admitted at the fictive ward unit (on imagined date 18th January)

**Room**	**Patient**	**Diagnose/Problem**	**Most recent events**	**Current plan**
510	Thomas Anderson, 03.12.82	Abdominal pain	Normal blood test results. Normal abdominal ultrasound.	
511	Mary Benson, 12.04.76	Colon cancer	Ultrasound kidneys 15th Jan.	Operation 20th Jan.
512	Monica Lot, 27.06.46	Pancreatitis	MRCP 19th Jan. Control CRP and Amylase every day.	Drainage
513	Janet Marsh, 13.12.44	Cholangitis	Has a urinary catheter.	Ampicillin i.v.
514	Gerda Dempsey, 05.02.38	Crohn	TPN since 13th Jan. CT 14th Jan.	Ordered gastro-enterological consult.
515	Mark Henderson, 30.08.64	Appendicitis	Operation 18th January	Abdominal X-ray 18th Jan. Can eat from 9 pm.
516	Oliver Hansson, 31.01.50	Abdominal pain; chest pain	Is at the moment at the imaging department for chest X-ray.	Fasting, awaiting blood test results and imaging.
517	Gabriel Veron, 19.05.23	Rectal bleeding	Known AAA. Gastroscopy 17th Jan.	Operation.

**Table 3:** Timeline for workflow and information updates during the demonstrations

**Simulated duration**	**Workflow and information updates during the demonstration**
10 min	Handover meeting
	#1: Sodium (Na) and potassium (K) results for Oliver Hansson’s blood test were ready. Both levels were within reference ranges (normal results). Gabriel Veron was at the operating room and his operation had started.
5 min	Visit patient room
	#2: No new information
5 min	Visit patient room
	#3: Troponin and haemoglobin results for Oliver Hansson. Both within reference ranges. Other laboratory results were expected to be available any minute. Chest X-ray was expected within 5 minutes.
10 min	Visit patient room
	#4: Chest X-ray and radiology summary report of Oliver Hansson were available. More laboratory results were also available (ALAT, GT, Platelets, WBC, CRP). Both the radiology report and several of the laboratory results were abnormal. Gabriel Veron’s operation was to be finished within 30 minutes.
10 min	Visit patient room
	#5: A referral for urgent surgery was available for Oliver Hansson. Additionally, a nurse had written a comment on Mark Henderson.

#### Evaluation

After each experiment a focused interview with each participant was carried out. The main topics of the interview were: Information needs, level of information details, information overload, future events, expected effects, authentication mechanisms, patient privacy, and re-identification based on contextual knowledge. Interviews were facilitated by having printed copies of the prototypes available (with varying levels of de-identification and abstraction), or by using the digital whiteboard actively for recapitulating the scenario and exploring functionality. All interviews were either audio or video recorded. The audio from all recordings was transcribed verbatim and analysed qualitatively in accordance with *systematic text condensation* (STC), a descriptive approach that focuses on the participants’ experiences as expressed by themselves, rather than interpretations of any underlying meaning of what they say [30]. STC shares many commonalities with other qualitative analyses, but has been developed to offer the researcher “a process of intersubjectivity, reflexivity, and feasibility, while maintaining a responsible level of methodological rigour” [30] (p. 795). We adhered strictly to each of the four distinct steps in STC: 1) Overviewing: The complete data set was read to establish preliminary themes based on the general impression of the data. 2) Coding: All meaning units were identified and coded. Meaning units were isolated sentences or sections of the text that somehow addressed the research questions. The codes given to these units were explicit and de-contextualised to enable grouping of similar meaning units. 3) Condensing: The meaning from each group of meaning units was abstracted. In this phase some codes needed adjustments to be able to make condensates reflecting all aspects of the meaning units. 4) Synthesising: In this final step, consistent and re-contextualised descriptions of the data were created. These descriptions should reflect the contents of the data and address the research questions, and were compared and validated against their context from which they had been derived (i.e. the raw textual material). The STC method was hence followed rigorously by both authors who read and coded the complete data set separately, in order to widen the analytic space and arrive at an increased understanding of the interview data [30,31].

### Ethics

All participants were introduced to the objective of the study and the methods both by information letter and verbally before the simulation began, and written consent forms had to be filled out. The image in Figure 1 was obtained for illustration purposes with written consent given by those pictured for its publication. The research project was approved by the Data Protection Official for Research for Norwegian universities.

## Results

The participants found the clinical scenario to be realistic. All interviews shared a common focus on the digital whiteboard and its use of de-identified/abstracted information. The qualitative analysis of the interview data resulted in six main themes: 1) The digital whiteboard as a medium; 2) patient privacy; 3) contextual re-identification; 4) information value; 5) expected effects; and 6) access control. Consistent descriptions pertaining to these themes are presented in the following subsections supplemented by selected quotations (translated to English). In general, the results are reported with a basis in the combined feedback from all three iterations. Some interview topics however, were only relevant for participants of certain iterations. The findings that are based on these topics are therefore reported with explicit reference to the iteration from which they were derived.

### Theme 1: the digital whiteboard as a medium

Participants appreciated being able to discover from a distance that new information regarding one of their patients was available. Sharing the same digital whiteboard with collaborating nurses was also regarded positive since they could discover new information for each other.

Nurse 6: “By a quick peek I saw that something had happened […]”

Nurse 8: “When you’re going to be in touch with patients as well, and apart from that have a bunch to do also, it is, as mentioned, incredibly neat to just take a quick glance at a screen, and go on like just: ‘OK’”.

Being able to access more detailed information through the same digital whiteboard (second and third iteration) was highly appreciated. Displaying updates about the patient, including concise medical information was regarded as one of the most important functionalities of the prototype. Quick access to actual blood test results and radiology summary reports–rather than only knowing that such information was available–was considered useful. While the same could apply to the other medical information as well, the participants did not see a justified need for accessing complete patient history on a digital whiteboard. Some had experienced that computers at the ward were often occupied, and feared that adding more detailed information to the digital whiteboard (e.g. long EHR notes) could result likewise.

Nurse 5: “Blood test results, imaging, referrals that have been sent–that’s okay. For instance that a medical consult has been ordered–that’s okay. (…) If you’re sort of going to study them closely, it’s more logical to sit down by a desktop computer and do it”.

Participants pointed out that the digital whiteboard should be visible in areas where they spend most of their time. In general the size of the digital whiteboard was regarded to be appropriate. Some, however, argued that smaller size would be better in terms of patient privacy, while others argued that it would be hard to hide the digital whiteboard entirely from passers-by and at the same time retain its visibility and usefulness for those working there. Ensuring that the digital whiteboard’s contents would not compromise patient privacy was considered more fruitful than having it located in a restricted area.

### Theme 2: patient privacy

With the highest level of de-identification applied (e.g.: “One of the patients has a new laboratory test result”), the participants said they would be comfortable with having the digital whiteboard located almost anywhere. Specifying the information presented–and not the patient–could also be acceptable (e.g.: “One of the patients has new results for sodium level analysis”). This could, however, depend on what kind of test that was specified. Although the digital whiteboard would not disclose neither the patient’s identity nor the actual test results, participants would in particular be less comfortable with displaying the availability of an HIV-test than they were with more common tests such as sodium.

In general, participants did not regard direct patient identifiers such as name, initials, or birth-year as viable alternatives due to their interpretation of privacy legislation. Room numbers could be accepted if the event category was presented alone without further specification (e.g. “A blood test result for patient in room 518 is available”). One remark, though, was that patients could easily find content having their own room number, and that this could be intimidating if patients were unaware of the limitations for what is shown on the digital whiteboard. Random pseudonyms were considered safe and precise (e.g. “Patient X24B has a new blood test result”), but also slightly ineffective, difficult and non-intuitive to use. In addition, they would most likely require introducing written lists with pseudonyms and corresponding patient names, and thus constitute a new risk for unwanted disclosure if misplaced or lost.

Nurse 4: “No, I would be sceptical to including that [birth-year and initials of patient]. It is better to log on the computer. You have to follow up on that information, anyway, but of course for me personally it would have been useful to know which patient that new information pertained to. But patient privacy-wise it is probably not completely acceptable. Patients can see each other in the corridors, and they realise who is old and young, and may understand who that information pertains to”.

Some participants commented that our approach with de-identification would respect patient privacy better than their current analogue whiteboards. Nevertheless, whenever accessing detailed event information, the participants emphasised the necessity of controlling what could be seen over their shoulder (third iteration). Patients, especially, could be looking through open doors. Hence, participants suggested smaller font size and minimising the area of the digital whiteboard where sensitive information would be presented. Additionally, the digital whiteboard should not be used for presenting text that would require prolonged reading.

### Theme 3: contextual re-identification

With a minimum of practice, the participants were able to see whenever new information was available on the digital whiteboard. Most often they were also capable of re-identifying the de-identified patient, even if no explicit patient identifiers were disclosed (e.g. “one of the patients at this ward has taken new X-Ray images”). While this ‘capability’ could be caused by the design of our simulated clinical scenario, the participants commented that they usually know what their patients have undergone and that they have expectations to what information and activities they are awaiting. There could be problems with the accuracy of such re-identification though, for instance if several patients have similar problems and synchronous care plans.

Nurse 1: “With eight patients at my unit, I will understand who that information relates to. (…) Patients are never operated the same time. (…) I would probably have checked it out anyway if I was uncertain about whether the patient was mine or not”.

Even if the participants did not understand which of their patients the information pertained to, they considered the digital whiteboard as useful since it could reduce amount of wasted checking for not yet available information.

Nurse 2: “The overview without names is better than having a black screen. We see whether something new is there that may be important to check; if something is going on–because there might be long periods when nothing happens. We see that it hasn’t happened, and thus we don’t have to log on when no new information is available”.

An idea of contextualising each piece of information with the clinician who produced the information, or the location where that information was produced, was not considered useful (first iteration). Either such information would not provide any additional distinction between patients, or it would not intersect with the recipient’s knowledge about the patient’s history. However, one of the participants suggested that new information could be labelled with the name of the responsible nurse, rather than any patient identifiers or room numbers. In that way the nurses would know when the information would be relevant for them.

Nurse 10: “Yes, for instance, they could be labelled with the responsible nurse above there. I don’t care, really, as long as I understand that they’re mine. Something that tells me they’re my patients, and that can just as well be my name instead of room numbers, actually”.

### Theme 4: information value

In general, information that would require some kind of follow-up action was most valued, e.g. being informed when new blood test results or radiologic summary reports were available, knowing when the patient should be pre-medicated before an operation, or when the patient should be transported to the operation room (OR) and back from the post-anaesthesia care unit. Likewise, knowing that an operation had ended was considered less valuable, since that did not prompt any particular action by the ward nurse. However, that information could be useful in another sense, i.e. informing the relatives of the patient about the progress in the OR. Importantly, the participants pointed out that time critical information should not be presented solely on the digital whiteboard without other measures as well. The nurses could be pre-occupied and not see the digital whiteboard for some time, hence requiring more direct communication.

Projections of future events and activities were highly valued. Knowing what the next step would be was considered useful both for planning of their own activities (including preparing patients more timely) and for verifying the current status of the patient.

Nurse 1: “This is great! For instance, regarding imaging, we don’t have an overview over imaging referrals or scheduled appointments, or if there has been any rescheduling. In particular, a patient that is scheduled for abdominal CAT-scan is supposed to drink contrast before the scan, and we often must call the imaging department and ask when the scan is scheduled because the patient is supposed to fast four hours before that. If that information was here, I think it would spare us much work”.

The accuracy of expected future events had to be reliable, yet did not necessarily need to be perfect. Some reckoned having estimates that were a bit too short were better than the opposite, and that continuously updated estimates could improve communication within the hospital.

Nurse 1: “We’re at a hospital, so you have to accept everything. Nothing turns out the way it was planned for (…) If the screen states that the X-ray is scheduled to be taken 11:00 am and the clock is 11:30 am when it gets done, that’s no crisis. But perhaps I would have phoned and asked ‘what is it with this delay?’. But they could as well have updated the time on the screen (…) That would be a nice way of communicating–just updating the screen. We wouldn’t have to make phone calls all the time”.

### Theme 5: expected effects

Participants explained that their current information systems did not provide any indications as to when new information would be available, so repeated checking was obligate. Hence, they expected that a system similar to the prototypes would reduce work-load, ease recognition of new information, and possibly speed up patient management. Although most information would still have to be communicated through phones or EHR, the participants expected reductions in the number of log-ins and log-outs in the EHR, improved overview of patient care activities, more proactive coordination of care and improved communication with patients and their relatives.

Nurse 6: “We spend quite much time logging in and checking for new blood test results and imaging reports, so we would save a lot of time there”.

Nurse 14: “ (…) when you get used to using this digital whiteboard, and get used to the colours and what it means, I believe this would be great to work faster; get faster aware of things”.

### Theme 6: access control

Participants wanted to be able to identify patients with confidence and access concise medical information related to each event that appeared on the digital whiteboard. The approach of disclosing identity and details by swiping an ID-card and entering a PIN-code was considered quick and simple, without substantial add-ons to their current workload (third iteration). However, if the card would have to be inserted somewhere, rather than swiped, some argued that ID-cards would be less convenient.

Nurse 10: “At a first glance I saw that something had happened without entering the PIN-code. And if it regards my patient and is interesting for me, I would naturally log on and see more”.

Nurse 11: “And if it is possible to swipe your card and enter your PIN-code just like we already do all day long wherever we are, then I think that would be great”.

In general, participants would accept user authentication measures as long as they were perceived proportional to the information they got access to. The authentication approach based on receiving sensitive information via SMS on a phone, was in this respect considered satisfying, but not optimal (second iteration). One advantage, though, was that the phone could be brought along for reading the information elsewhere. On the contrary, it required much effort sending and then browsing through several messages, and a lost phone could reveal sensitive information if not protected by additional authentication.

Biometric techniques such as fingerprint, iris scanning or facial recognition through video capture were questioned during the interviews (second and third iteration), but not tested in practice. The participants said that such methods could work, but their implementation had to be solid, quick, and not cause any additional clutter for the user.

The participants agreed on the need for an automatic mechanism for logging out (second and third iteration). Some told that they sometimes did not log out when leaving a computer, for instance in emergency situations or due to time-consuming authentication mechanisms in their current systems. Specifying a particular time limit for automatic logout was challenging, and the participants’ suggestions varied between ten seconds and two minutes–balancing patient privacy with annoying unintended session time-outs.

Nurse 9: “If the screen is inactive for so and so long and then it logs off automatically, that would of course be a kind of must-have. Because suddenly something happens, and you’ve just got to run without considering the fact that you are still logged in”.

Nurse 12: “Now, imagine I’m a little busy, but still in front of the screen waiting to take a look at it, and then I talk to someone. And then, when looking at the screen again I’m logged out again. That’s very annoying!”

## Discussion

### Summary of main findings

This study demonstrates how professional ward nurses can exploit patient centred, de-identified and abstracted clinical information presented on a digital whiteboard for coordination of care. The study indicates that ward nurses might be capable of re-identifying de-identified information based on their knowledge of their patients’ recent and future care activities. However, even if not completely able to do so, de-identified information has the potential of reducing work-load by reducing the number of log-ins to the EHR. This includes log-ins that could very well be avoided, such as simple checks for availability of new information. Potentially, the nurses could also become more rapidly aware of new information. In addition to displaying recent clinical events on a digital whiteboard, the nurses could benefit from having estimates of future events as well. Given a sufficient level of reliability of such estimates, this information might improve communication and coordination of work, according to the nurses. Future events may also aid the nurses in contextualising de-identified/abstracted information.

These results also indicate how combinations of privacy enhancing design techniques can provide a level of patient privacy that is acceptable while maintaining a useful digital whiteboard system. However, nurses should also be able to verify patient identity and access more detailed medical information with little additional effort. If such disclosure is done on a digital whiteboard, it is important for the user to have effective control over what can be seen by patients and passers-by at any time.

### Strengths and limitations

Our findings are based on the subjective experience and expectations of professional ward nurses after using a prototype in a simulated ward setting. This study design obviously does not provide any final conclusions on neither the usability of the chosen design features, nor on the effects of an implemented system at a real ward. Still, we consider our findings as both relevant and important as input for understanding ward work and developing support for coordination of care.

The clinical scenario in our experiments was designed with two purposes in mind: First, to provide a realistic context for the prototype, and second, to demonstrate the functionalities of the prototype. Hence, the scenario also reflected the preconceptions of the researchers. According to the participants we succeeded with creating a realistic clinical scenario. To avoid verifying our preconceptions during the interviews we emphasised participants’ opinions on the effects and applicability of such a system during a typical day at work. We asked them to exemplify their opinions with descriptions of situations from actual ward work. Thus, we took advantage of their clinical experience rather than asking how they valued the prototype as part of our simulated scenario. Variations in auxiliary technology and authentication mechanisms between the three iterations might also have affected the participants’ perceptions of the prototypes. In particular, this limits the validity of our findings related to access control, since these mechanisms varied the most between the iterations and were hence not demonstrated for all participants. Apart from that, we do not think this has biased our findings, considering that demonstration sessions were a means for focusing the subsequent interview. We recall that the purpose of doing focused interviews was to explore information needs in this particular domain, rather than evaluating the usability of each prototype. In our opinion, the different professional backgrounds of the two researchers carrying out the qualitative analysis also contributed to an improved understanding of the data. Having undertaken these measures, as well as having adhered to the methodological rigour of STC, we consider the internal validity of our findings to be good, and the confounding effects of the researchers’ preconceptions on these findings to be sufficiently low. The chosen sample does however not permit uncritical and direct generalisation of the results to other ward settings. It is very likely that the findings are related to experience of the ward nurses and their organisation of work (e.g. who is responsible for following up blood test results). Furthermore, the number of patients presented on the digital whiteboard is most likely a critical factor, as fewer patients would decrease patient privacy and more patients would make it more difficult for the nurses to contextually re-identify to whom new information pertains. Nevertheless, conceptually, some of these results may be transferred to other settings, e.g. increasing visibility of de-identified and abstracted new clinical information, or providing clinicians with estimates of future clinical events, at least as interesting hypotheses that could and should be followed-up in future studies. Finally, it must be kept in mind that novel technology and organisational changes might introduce unexpected side-effects to current work-flow and care. This has not been investigated sufficiently in this study.

### Supporting coordination of care

Previous studies of digital whiteboards and computerised patient tracking systems have found that such technology may improve quality of data [18], increase patient satisfaction [16], and improve communication and coordination of care [10,17-19,32]. Financial, administrative, educational and research benefits also add to the positive effects of such technology [15]. However, others have experienced negative effects, such as increases in coordination breakdowns [33] and less accurate information compared to dry-erase whiteboards [13].

A recent systematic review of 21 studies of digital whiteboards situated in hospital departments intended to answer what consequences such technology has on work, and what mediating factors influence these consequences [34]. However, the results of the review were mixed, and the author concluded that more studies “into the areas of display format, interface design, integration to other systems and user involvement seems relevant in order to increase our knowledge regarding the development and implementation of electronic whiteboards” [34] (p. 491). Overall, the participants in our study had a positive attitude towards our prototypes, and expected positive effects on coordination and inter-departmental communication. This, obviously, does not serve as proof of actual consequences of such technology on hospital work, but the results from our involvement of intended users in formative evaluation of tangible artefacts is–in our opinion–a significant contribution to the advancement of this field of research and development.

A qualitative study of nurse coordinators at a high-volume trauma hospital found that information tools that are meant for supporting communication and care coordination should meet some specified design goals in order to fit into the nurses’ tasks; “(1) making information compatible with the mobile nature of their work, (2) enabling rapid information access and note-taking under time pressure, and (3) supporting rapid information processing and attention management through the effective use of layout design, shorthand symbols, and color-coding” [35] (p. 667). According to what the participants in our experiments explained, our prototypes met most of these design goals. However, differences in such mediating factors between implemented versions of digital whiteboards might be one explanation for varying research findings. Another explanation might be that the perceived effects may vary between staff groups and evolve over time [11].

Deciding what information should or should not be included on a digital whiteboard for supporting coordination of care is not straightforward. On the one hand, participants said that information that did not require a particular follow-up was of less value to them, but on the other hand, information that needed immediate follow-up should not be presented solely on a digital whiteboard (e.g. critical laboratory results). Based on our results, we have no definite answer to neither what is the optimal set of information nor how to arrive at that set of information. However, for ward nurses in a comparable organisational setting, our findings give a direction to what might be more important to include. For other purposes, developers could involve actual end-users in similar simulations as we did or other kinds of participatory workshops. Another alternative is to develop technology that permits individual subscription to information each user regards as important. This, of course, would require more research and development.

Our participants were positive to having continuously updated projections of future clinical events, including events for which no schedule typically has been available. While the ability to project the future situation has been considered to be the highest level of situation awareness [5], and predictive aids have been shown to be valuable in aviation and other domains [6,36], this has not been the main focus in the literature on digital whiteboards for coordination of care. Some exceptions exist, though, such as the continuously updated operating room schedule referred to as “AwareMedia” [20]. Like our prototypes, AwareMedia presents a continuously updated log of past and current activities as they unfold, as well as the “anticipated future flow of work” [20] (p. 110). Both AwareMedia and our prototypes cope with the uncertainty of future activities by continuously updating the projection. Another strategy is to inform the user about the known uncertainty [37]. However, this possibility was not explored in our study. Our participants stated that accuracy had to be reliable, but not perfect. We would like to hypothesise that with modern technology it is possible to make such predictions in real-time with sufficiently high reliability and accuracy (at least for certain activities such as automated laboratory analyses). We think this should be further studied, both with and without any techniques for visualising temporal uncertainty. In our opinion, the possible effects of being able to predict quite accurately the next 60 minutes of work, or perhaps even less, in a complex institution such as a hospital, should not be underestimated.

### Balancing visibility against patient privacy

How to balance patient privacy against user requirements and legislative requirements is another aspect of shared digital whiteboards that has rarely and barely been focused on in the context of coordination of healthcare activities. While Scupelli et al. [24] suggest to place whiteboards in staff-only areas to avoid leaving out information due to privacy legislation, Aronsky et al. [15] ask patients to sign a waiver for displaying their names on the whiteboard, and include screen savers, aggressive time-outs and authentication for enhanced privacy protection. Bardram and Bossen [38] have suggested authentication mechanisms based on physical proximity, and like us, they argue that “some information and some views on this information may not be subject to user authentication”. Theoretically, there could be several levels of security/privacy needs depending on the importance and quality of the disclosed information, physical access control and surroundings, and time of day [39], in addition to available functionality (i.e. read-only or also write/edit access). This indicates that the common “all or nothing” approach should be reconsidered for access control implementations in digital whiteboards. While authentication of users can be done by something the users either *know* (e.g. a password), *have* (e.g. a smart card) or *are* (e.g. fingerprint, iris pattern or other biometrics), it was not the scope of this study to explore all these techniques in detail. Flexible de-identification is even based on something the users *recognise*. Studies have nevertheless shown that common access control implementations can pose a threat to security in collaborative environments, e.g. clinicians may share passwords or access with colleagues or avoid logging out if they do not understand the user interface or if security measures do not match their needs [40,41]. This emphasises the importance of a holistic approach to privacy and information security design for digital whiteboards, taking the end users’ actual usage scenarios into account from the start. Rather than focusing solely on authentication, focus should be shifted towards security as a combined product of the interactive system and the people who use it [42]. It might be fruitful to develop systems that allow users to “understand the consequences of their actions and develop new forms of practice” [43], although it has been argued that it may be risky to expect this from users [21,29]. The results from our third iteration indicated that the nurses wanted to control what can been seen over their shoulder while interacting with the digital whiteboard. The participants suggested various methods for increased control: Reducing the font-size (complicating perception from distance), limited areas for disclosure (enabling blocking of contents by the user’s hand or body) and in general avoiding information that requires prolonged reading (reducing availability of sensitive information). The idea of disclosing and hiding only limited areas of the screen, as it was implemented in the third iteration as a variation of small pop-up windows, resembles what has previously been referred to as ‘privacy blinders’. Although using this may require more effort compared to having no blinders, it can still be accepted if it provides “levels of privacy that would otherwise not exist” [44].

An alternative to privacy blinders is coding of information with colours, shapes, etc. [44]. Users are able learn how to use such codes. However, in itself such ‘security by obscurity’ is not a secure approach since codes always run the risk of being decoded by others. Although we used colour coding of the event categories in our prototypes, our approach is fundamentally different from ‘security by obscurity’. Rather than coding information, we removed information details from the default view (all iterations) and introduced ambiguity in the identification of individuals (first and second iteration). Only the event categories were visible in the default view in the third iteration. These were colour coded, not to obscure information, but rather to make that information readable from a distance. Our participants did not consider visualising event categories room-wise as breaching privacy rights (e.g. “the patient in room 512 had an imaging event”). However, this probably depends on how broad the event categories are defined. Reintroducing ambiguity in identification of individual patients increases the level of security, e.g. by organising events per nurse rather than per patient room. This was also proposed as a potential usability improvement by one of the participants.

The use of mobile devices for accessing more details is a security alternative that we only marginally have explored with this study, and hence we do not discuss that any further. Other aspects of privacy design related to policy, system and interaction issues in privacy sensitive systems can be found elsewhere [45]. To our knowledge, the usability of most of these design patterns have not been evaluated for shared whiteboard systems in hospital contexts. This calls for more research on the balance between patient privacy and visibility of clinical information.

## Conclusions

Displaying patient centred, de-identified and abstracted clinical events on a digital whiteboard is a promising technology for supporting coordination of care at a hospital ward. This study indicates that such technology has the potential to reduce workload for ward nurses, speed up patient management and enable a more proactive coordination of care–without compromising patient privacy. However, this privacy enhancing design approach remains to be applied and evaluated in real clinical work, and it is unknown whether other technological alternatives to digital whiteboards are more effective.

## Competing interests

Both authors declare that they have no competing interests to declare.

## Authors’ contributions

Both authors have contributed equally in designing the study, conducting the experiments and analysing the results. Both authors have revised all parts of the article critically for important intellectual content, and given their approval of the final manuscript. Hence, they both contributed equally to this work and share first authorship.

## Pre-publication history

The pre-publication history for this paper can be accessed here:

http://www.biomedcentral.com/1472-6947/14/27/prepub

## Supplementary Material

Additional file 1 Digital whiteboard prototype. Click here for file
