# Supplementary material for: Designing privacy-friendly digital whiteboards for mediation of clinical progress
Source: BMC Med Inform Decis Mak. 2014 Apr 4;14:27. doi: 10.1186/1472-6947-14-27 (PMC4021250; doi:10.1186/1472-6947-14-27)
Supplement: Additional file 1 — Digital whiteboard prototype. [file 1472-6947-14-27-S1.zip › index.html]

COSTTVIZ - English


|  |
| --- |
|  |
| Enter PIN code:  |  |  |  | | --- | --- | --- | |  |  |  | |  |  |  | |  |  |  | |  |  |  | |
| Identified as: **T. K. Goodlowe** Phone: 71324 |
|  |
|  |

**Authenticate**  
by touching  
ID-card here..
