# Supplementary figures and images for: Designing privacy-friendly digital whiteboards for mediation of clinical progress (part 2 of 2)
Source: BMC Med Inform Decis Mak. 2014 Apr 4;14:27. doi: 10.1186/1472-6947-14-27 (PMC4021250; doi:10.1186/1472-6947-14-27)

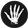

Supplement: Additional file 1 — Digital whiteboard prototype. [file 1472-6947-14-27-S1.zip › gfx/_radiology.png]

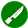

Supplement: Additional file 1 — Digital whiteboard prototype. [file 1472-6947-14-27-S1.zip › gfx/_surgery.png]

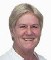

Supplement: Additional file 1 — Digital whiteboard prototype. [file 1472-6947-14-27-S1.zip › gfx/_user.jpg]

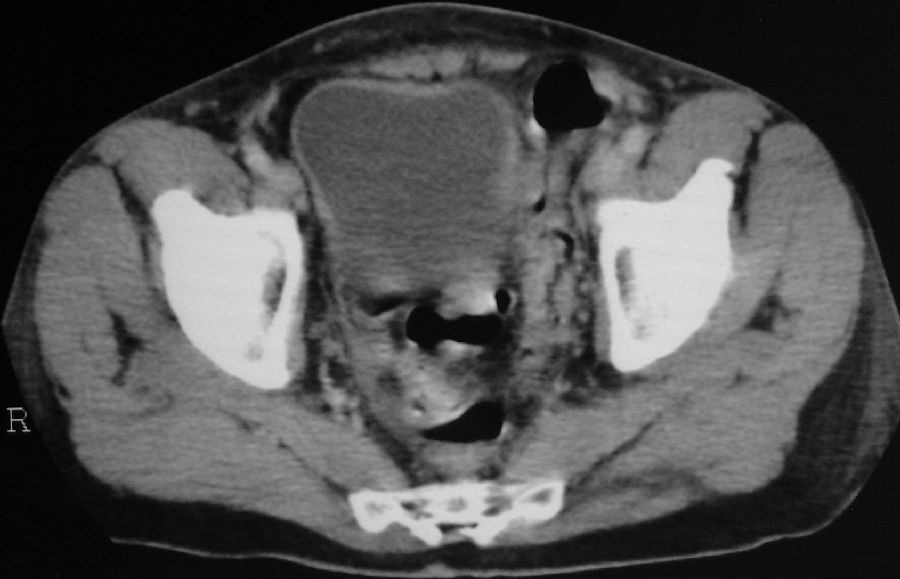

Supplement: Additional file 1 — Digital whiteboard prototype. [file 1472-6947-14-27-S1.zip › res/gerd1.jpg]

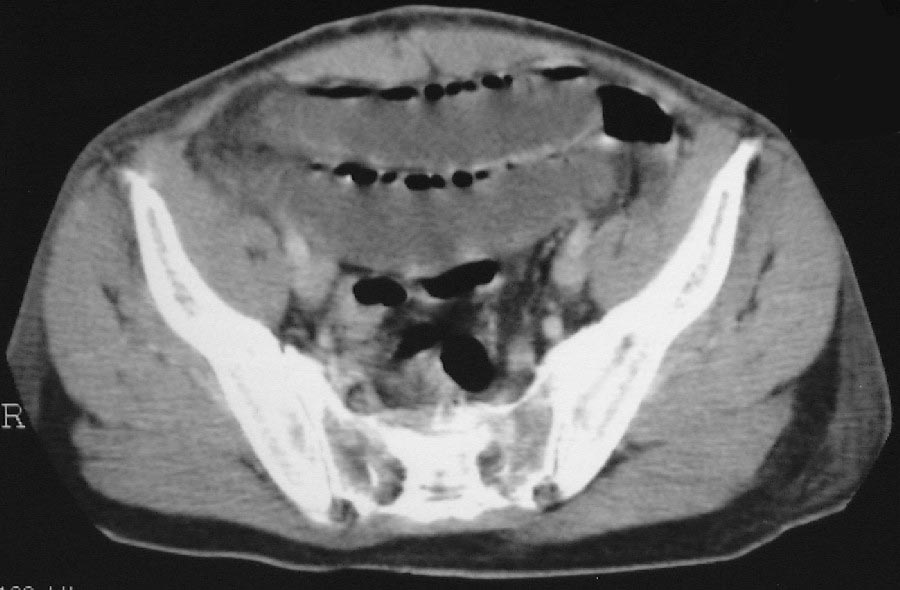

Supplement: Additional file 1 — Digital whiteboard prototype. [file 1472-6947-14-27-S1.zip › res/gerd2.jpg]

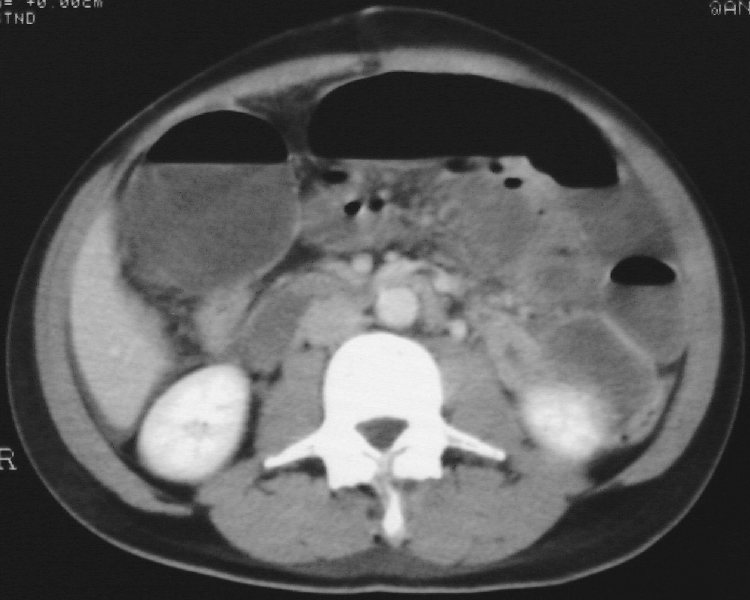

Supplement: Additional file 1 — Digital whiteboard prototype. [file 1472-6947-14-27-S1.zip › res/gerd3.jpg]

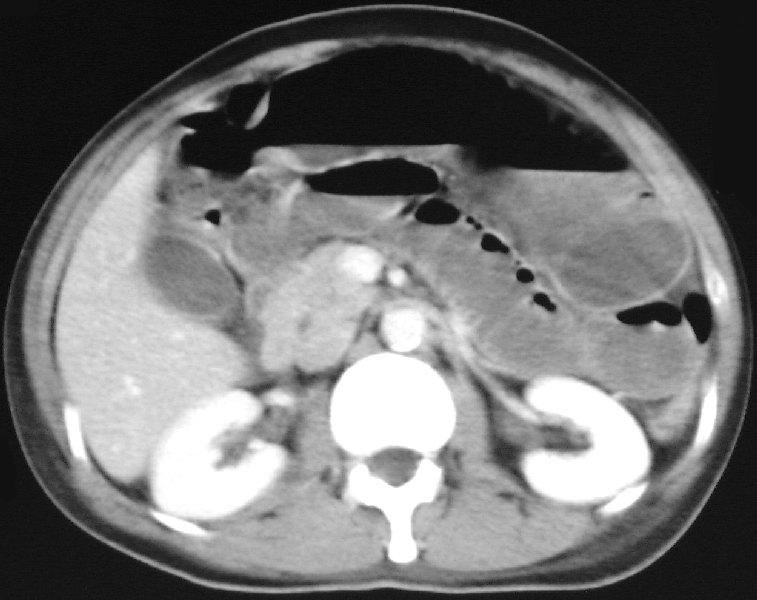

Supplement: Additional file 1 — Digital whiteboard prototype. [file 1472-6947-14-27-S1.zip › res/gerd4.jpg]

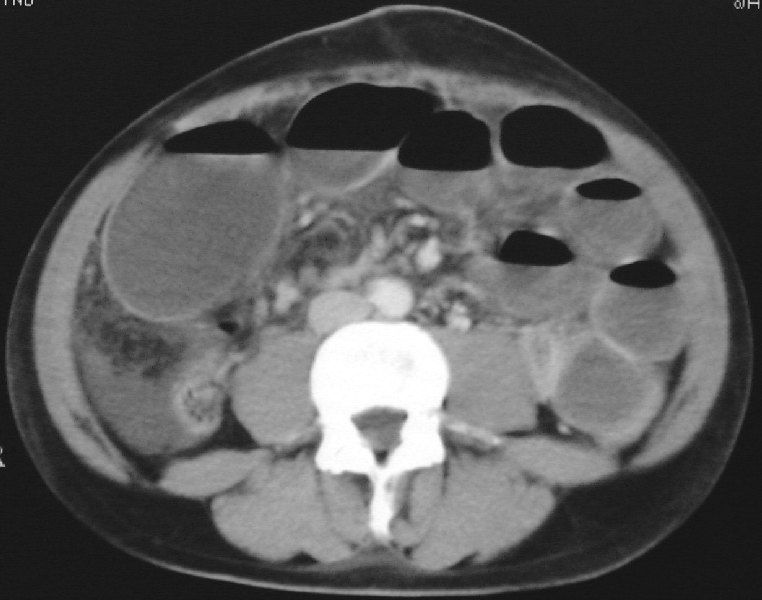

Supplement: Additional file 1 — Digital whiteboard prototype. [file 1472-6947-14-27-S1.zip › res/gerd5.jpg]

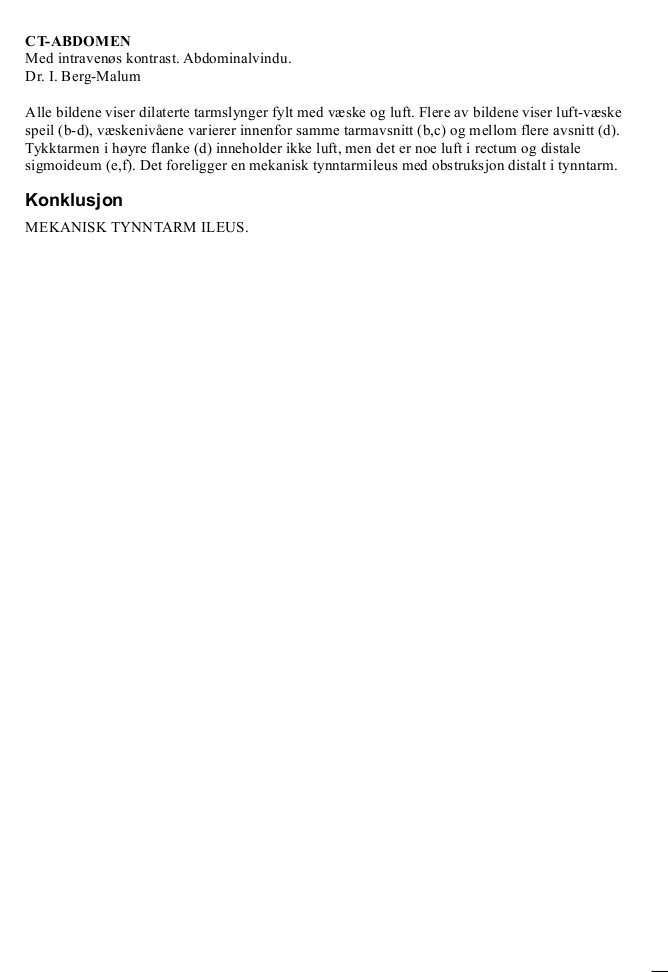

Supplement: Additional file 1 — Digital whiteboard prototype. [file 1472-6947-14-27-S1.zip › res/gerddahl_CT-beskrivelse.png]

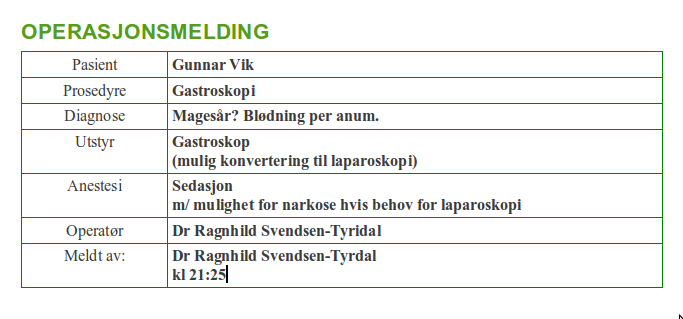

Supplement: Additional file 1 — Digital whiteboard prototype. [file 1472-6947-14-27-S1.zip › res/gunnarvik_operasjonsmelding.png]

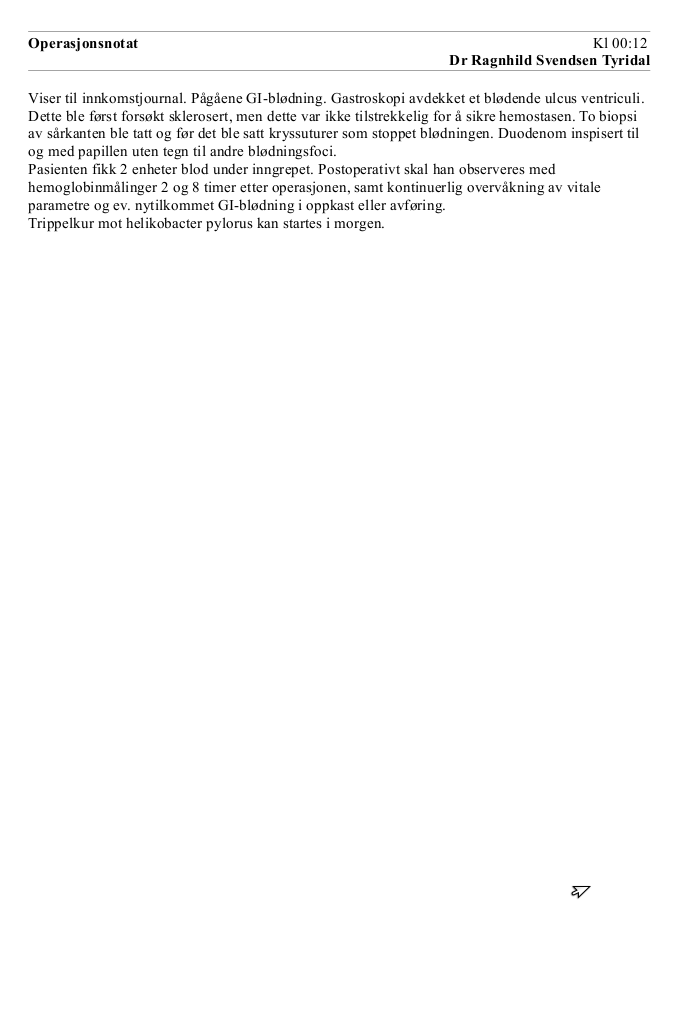

Supplement: Additional file 1 — Digital whiteboard prototype. [file 1472-6947-14-27-S1.zip › res/gunnarvik_operasjonsnotat.png]

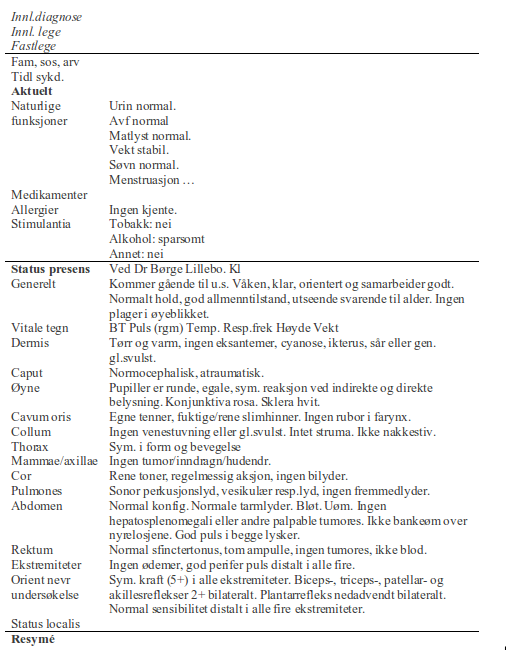

Supplement: Additional file 1 — Digital whiteboard prototype. [file 1472-6947-14-27-S1.zip › res/innkomstjournal_empty.png]

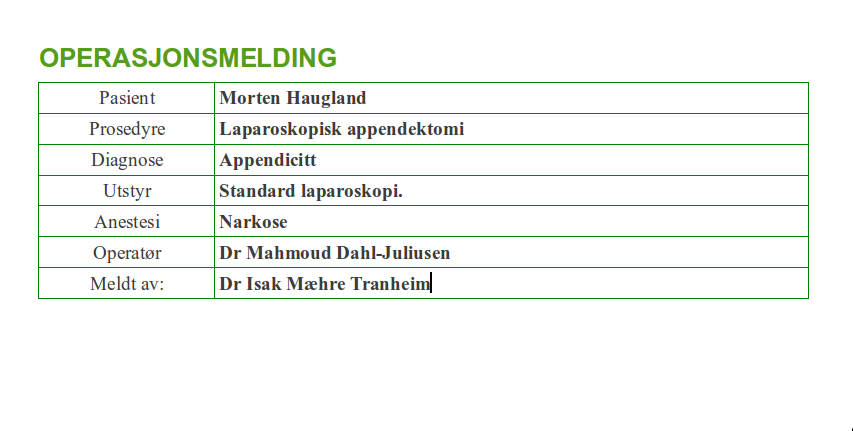

Supplement: Additional file 1 — Digital whiteboard prototype. [file 1472-6947-14-27-S1.zip › res/morten_operasjonsmelding.png]

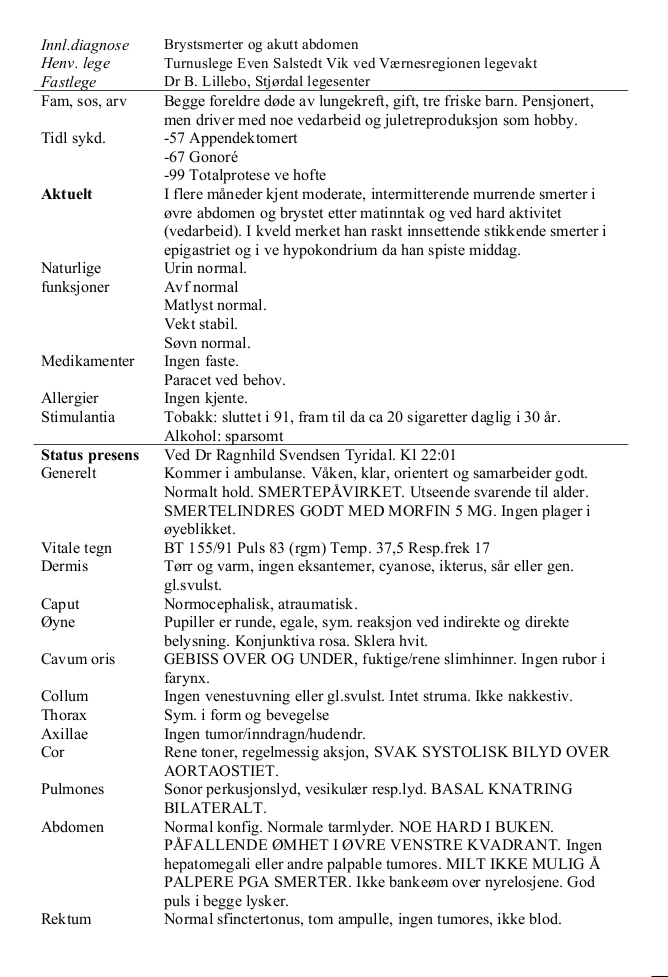

Supplement: Additional file 1 — Digital whiteboard prototype. [file 1472-6947-14-27-S1.zip › res/oddhansen_innkomst_1.png]

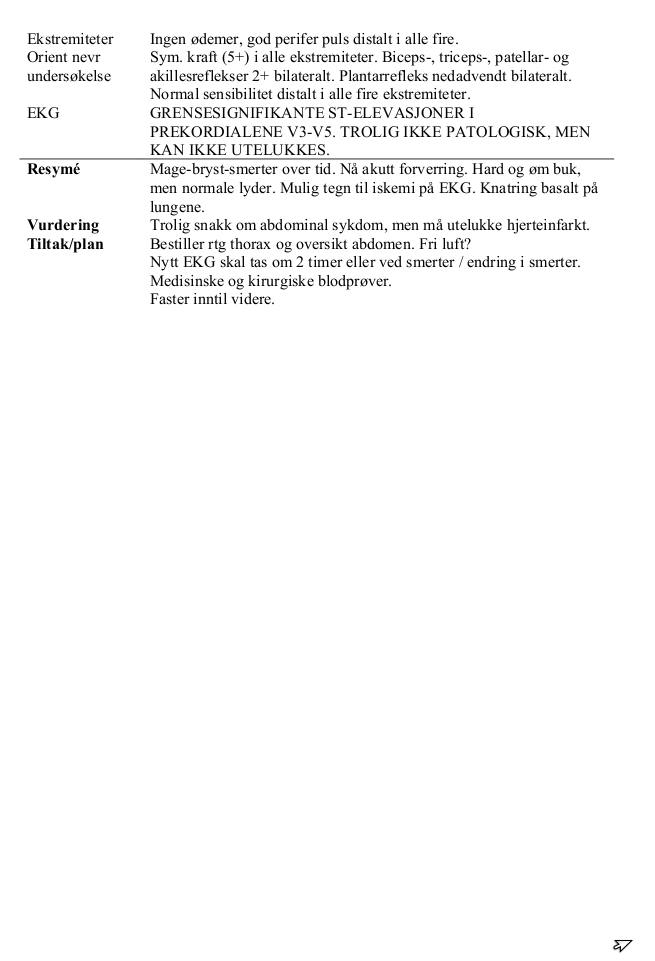

Supplement: Additional file 1 — Digital whiteboard prototype. [file 1472-6947-14-27-S1.zip › res/oddhansen_innkomst_2.png]

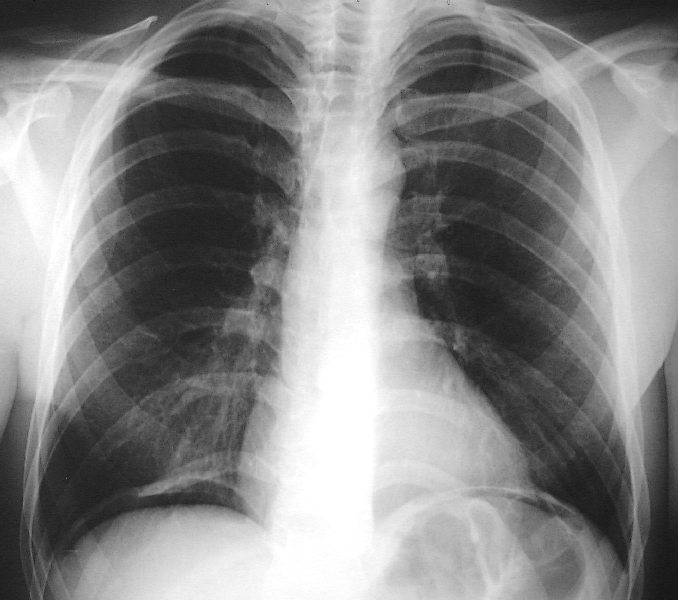

Supplement: Additional file 1 — Digital whiteboard prototype. [file 1472-6947-14-27-S1.zip › res/oddhansen_rtg1.jpg]

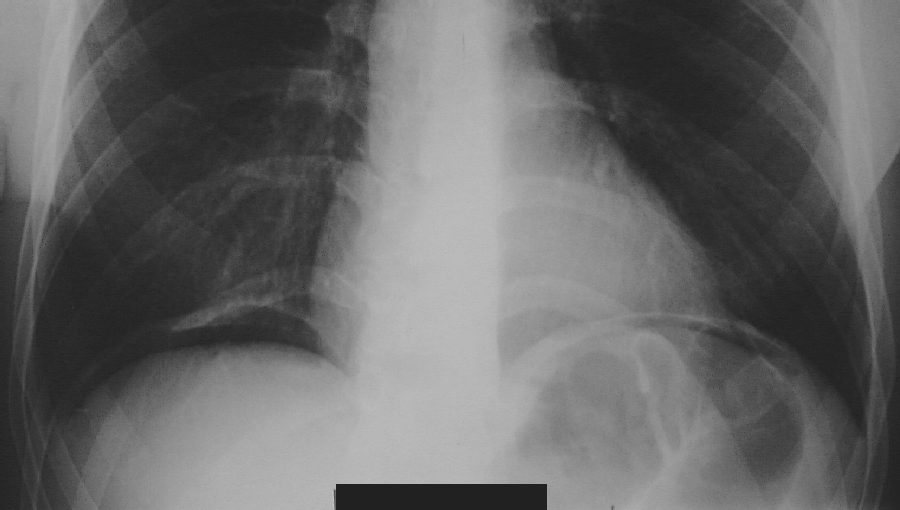

Supplement: Additional file 1 — Digital whiteboard prototype. [file 1472-6947-14-27-S1.zip › res/oddhansen_rtg2.jpg]

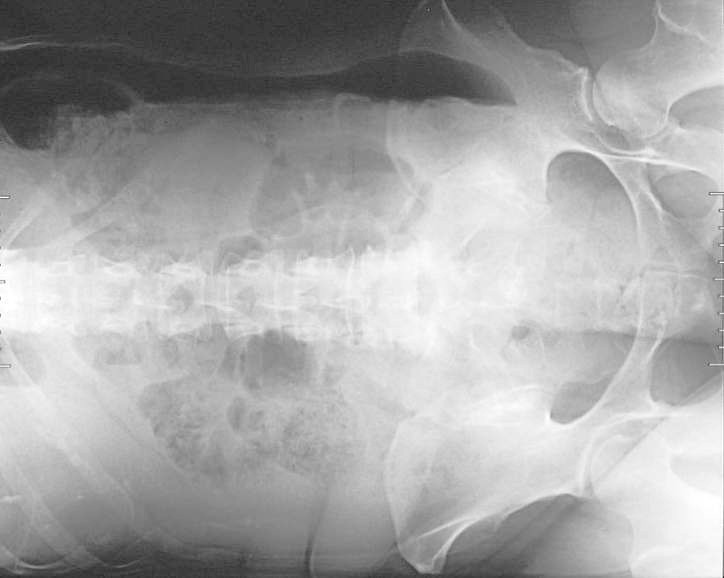

Supplement: Additional file 1 — Digital whiteboard prototype. [file 1472-6947-14-27-S1.zip › res/oddhansen_rtg3.jpg]

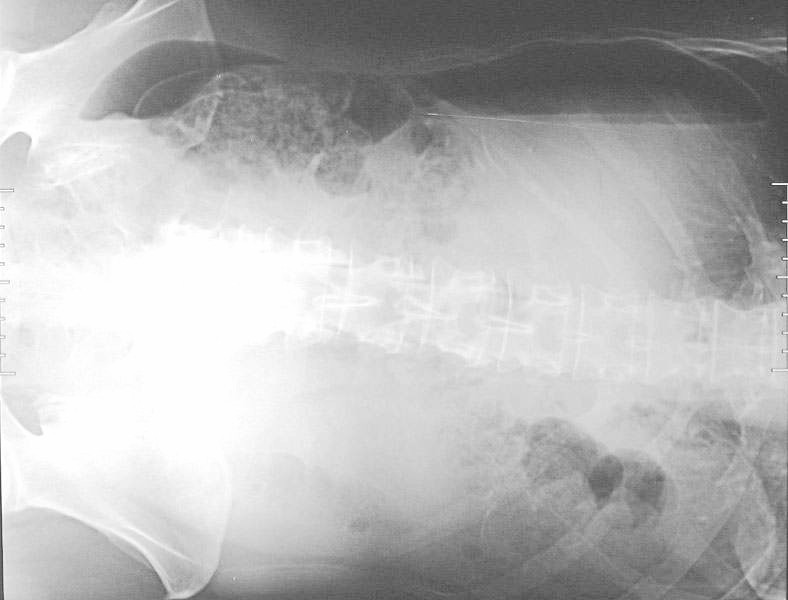

Supplement: Additional file 1 — Digital whiteboard prototype. [file 1472-6947-14-27-S1.zip › res/oddhansen_rtg4.jpg]

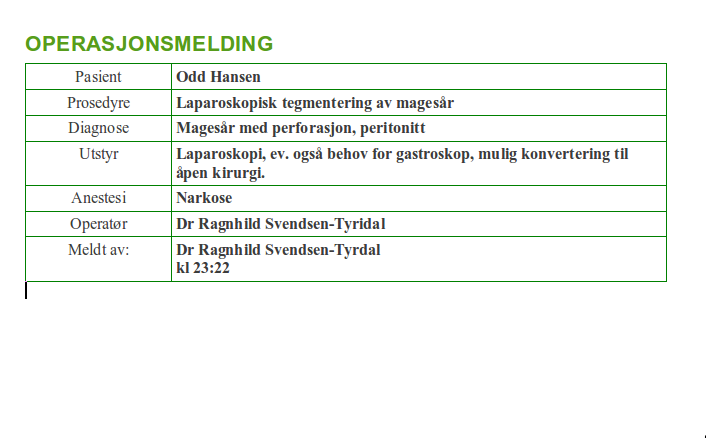

Supplement: Additional file 1 — Digital whiteboard prototype. [file 1472-6947-14-27-S1.zip › res/odd_operasjonsmelding.png]

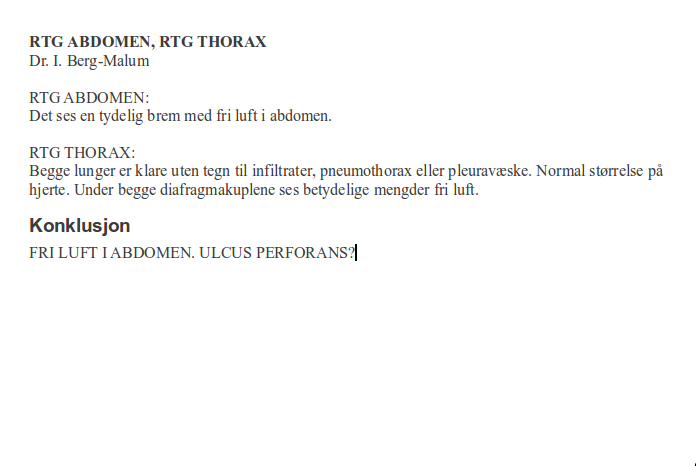

Supplement: Additional file 1 — Digital whiteboard prototype. [file 1472-6947-14-27-S1.zip › res/odd_rtgbeskrivelse.png]

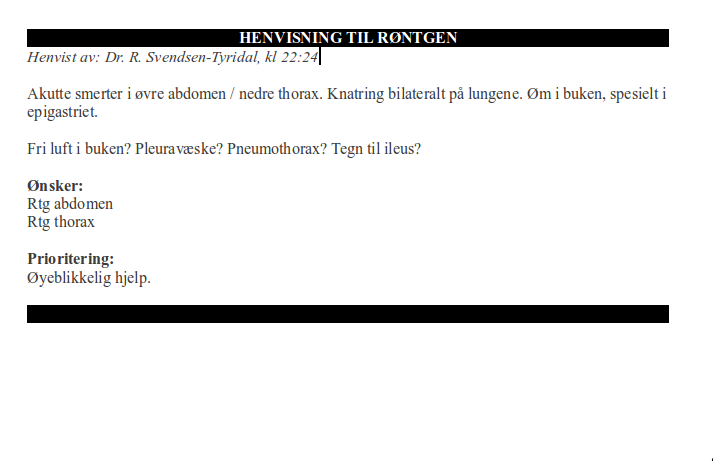

Supplement: Additional file 1 — Digital whiteboard prototype. [file 1472-6947-14-27-S1.zip › res/odd_rtghenvisning.png]

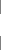

Supplement: Additional file 1 — Digital whiteboard prototype. [file 1472-6947-14-27-S1.zip › css/images/border.png]

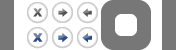

Supplement: Additional file 1 — Digital whiteboard prototype. [file 1472-6947-14-27-S1.zip › css/images/controls.png]

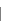

Supplement: Additional file 1 — Digital whiteboard prototype. [file 1472-6947-14-27-S1.zip › css/images/ie6/borderBottomCenter.png]

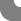

Supplement: Additional file 1 — Digital whiteboard prototype. [file 1472-6947-14-27-S1.zip › css/images/ie6/borderBottomLeft.png]

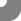

Supplement: Additional file 1 — Digital whiteboard prototype. [file 1472-6947-14-27-S1.zip › css/images/ie6/borderBottomRight.png]

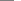

Supplement: Additional file 1 — Digital whiteboard prototype. [file 1472-6947-14-27-S1.zip › css/images/ie6/borderMiddleLeft.png]

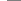

Supplement: Additional file 1 — Digital whiteboard prototype. [file 1472-6947-14-27-S1.zip › css/images/ie6/borderMiddleRight.png]

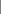

Supplement: Additional file 1 — Digital whiteboard prototype. [file 1472-6947-14-27-S1.zip › css/images/ie6/borderTopCenter.png]

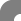

Supplement: Additional file 1 — Digital whiteboard prototype. [file 1472-6947-14-27-S1.zip › css/images/ie6/borderTopLeft.png]

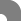

Supplement: Additional file 1 — Digital whiteboard prototype. [file 1472-6947-14-27-S1.zip › css/images/ie6/borderTopRight.png]

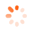

Supplement: Additional file 1 — Digital whiteboard prototype. [file 1472-6947-14-27-S1.zip › css/images/loading.gif]

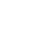

Supplement: Additional file 1 — Digital whiteboard prototype. [file 1472-6947-14-27-S1.zip › css/images/loading_background.png]

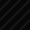

Supplement: Additional file 1 — Digital whiteboard prototype. [file 1472-6947-14-27-S1.zip › css/images/overlay.png]

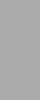

Supplement: Additional file 1 — Digital whiteboard prototype. [file 1472-6947-14-27-S1.zip › css/smoothness/images/ui-bg_flat_0_aaaaaa_40x100.png]

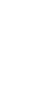

Supplement: Additional file 1 — Digital whiteboard prototype. [file 1472-6947-14-27-S1.zip › css/smoothness/images/ui-bg_flat_75_ffffff_40x100.png]

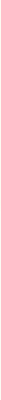

Supplement: Additional file 1 — Digital whiteboard prototype. [file 1472-6947-14-27-S1.zip › css/smoothness/images/ui-bg_glass_55_fbf9ee_1x400.png]

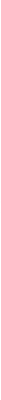

Supplement: Additional file 1 — Digital whiteboard prototype. [file 1472-6947-14-27-S1.zip › css/smoothness/images/ui-bg_glass_65_ffffff_1x400.png]

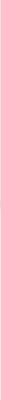

Supplement: Additional file 1 — Digital whiteboard prototype. [file 1472-6947-14-27-S1.zip › css/smoothness/images/ui-bg_glass_75_dadada_1x400.png]

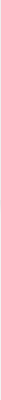

Supplement: Additional file 1 — Digital whiteboard prototype. [file 1472-6947-14-27-S1.zip › css/smoothness/images/ui-bg_glass_75_e6e6e6_1x400.png]

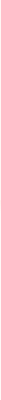

Supplement: Additional file 1 — Digital whiteboard prototype. [file 1472-6947-14-27-S1.zip › css/smoothness/images/ui-bg_glass_95_fef1ec_1x400.png]

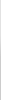

Supplement: Additional file 1 — Digital whiteboard prototype. [file 1472-6947-14-27-S1.zip › css/smoothness/images/ui-bg_highlight-soft_75_cccccc_1x100.png]

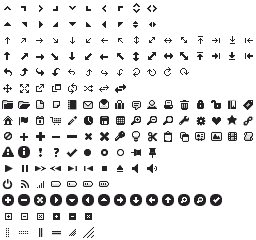

Supplement: Additional file 1 — Digital whiteboard prototype. [file 1472-6947-14-27-S1.zip › css/smoothness/images/ui-icons_222222_256x240.png]

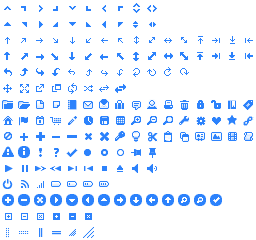

Supplement: Additional file 1 — Digital whiteboard prototype. [file 1472-6947-14-27-S1.zip › css/smoothness/images/ui-icons_2e83ff_256x240.png]

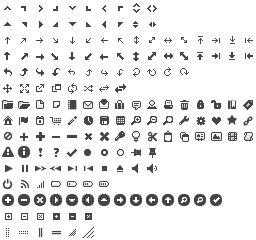

Supplement: Additional file 1 — Digital whiteboard prototype. [file 1472-6947-14-27-S1.zip › css/smoothness/images/ui-icons_454545_256x240.png]

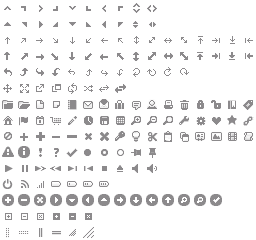

Supplement: Additional file 1 — Digital whiteboard prototype. [file 1472-6947-14-27-S1.zip › css/smoothness/images/ui-icons_888888_256x240.png]

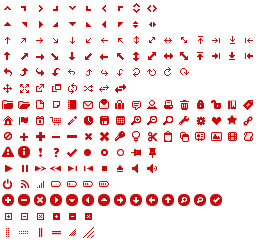

Supplement: Additional file 1 — Digital whiteboard prototype. [file 1472-6947-14-27-S1.zip › css/smoothness/images/ui-icons_cd0a0a_256x240.png]
