# Supplementary figures and images for: Designing privacy-friendly digital whiteboards for mediation of clinical progress (part 1 of 2)
Source: BMC Med Inform Decis Mak. 2014 Apr 4;14:27. doi: 10.1186/1472-6947-14-27 (PMC4021250; doi:10.1186/1472-6947-14-27)

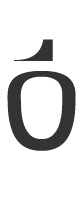

Supplement: Additional file 1 — Digital whiteboard prototype. [file 1472-6947-14-27-S1.zip › gfx/clock/0-1.png]

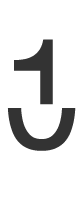

Supplement: Additional file 1 — Digital whiteboard prototype. [file 1472-6947-14-27-S1.zip › gfx/clock/0-2.png]

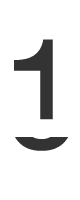

Supplement: Additional file 1 — Digital whiteboard prototype. [file 1472-6947-14-27-S1.zip › gfx/clock/0-3.png]

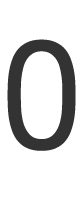

Supplement: Additional file 1 — Digital whiteboard prototype. [file 1472-6947-14-27-S1.zip › gfx/clock/0.png]

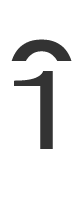

Supplement: Additional file 1 — Digital whiteboard prototype. [file 1472-6947-14-27-S1.zip › gfx/clock/001-1.png]

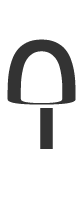

Supplement: Additional file 1 — Digital whiteboard prototype. [file 1472-6947-14-27-S1.zip › gfx/clock/001-2.png]

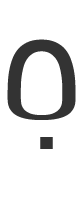

Supplement: Additional file 1 — Digital whiteboard prototype. [file 1472-6947-14-27-S1.zip › gfx/clock/001-3.png]

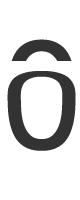

Supplement: Additional file 1 — Digital whiteboard prototype. [file 1472-6947-14-27-S1.zip › gfx/clock/01-1.png]

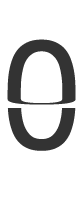

Supplement: Additional file 1 — Digital whiteboard prototype. [file 1472-6947-14-27-S1.zip › gfx/clock/01-2.png]

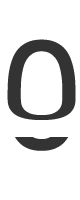

Supplement: Additional file 1 — Digital whiteboard prototype. [file 1472-6947-14-27-S1.zip › gfx/clock/01-3.png]

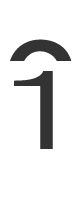

Supplement: Additional file 1 — Digital whiteboard prototype. [file 1472-6947-14-27-S1.zip › gfx/clock/1-1.png]

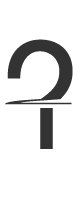

Supplement: Additional file 1 — Digital whiteboard prototype. [file 1472-6947-14-27-S1.zip › gfx/clock/1-2.png]

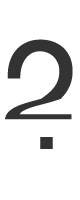

Supplement: Additional file 1 — Digital whiteboard prototype. [file 1472-6947-14-27-S1.zip › gfx/clock/1-3.png]

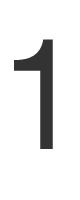

Supplement: Additional file 1 — Digital whiteboard prototype. [file 1472-6947-14-27-S1.zip › gfx/clock/1.png]

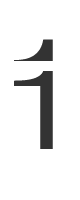

Supplement: Additional file 1 — Digital whiteboard prototype. [file 1472-6947-14-27-S1.zip › gfx/clock/11-1.png]

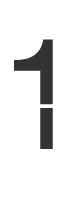

Supplement: Additional file 1 — Digital whiteboard prototype. [file 1472-6947-14-27-S1.zip › gfx/clock/11-2.png]

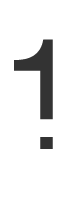

Supplement: Additional file 1 — Digital whiteboard prototype. [file 1472-6947-14-27-S1.zip › gfx/clock/11-3.png]

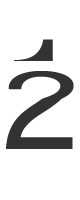

Supplement: Additional file 1 — Digital whiteboard prototype. [file 1472-6947-14-27-S1.zip › gfx/clock/111-1.png]

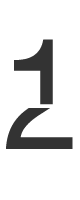

Supplement: Additional file 1 — Digital whiteboard prototype. [file 1472-6947-14-27-S1.zip › gfx/clock/111-2.png]

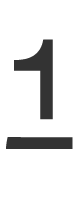

Supplement: Additional file 1 — Digital whiteboard prototype. [file 1472-6947-14-27-S1.zip › gfx/clock/111-3.png]

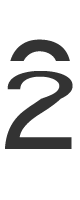

Supplement: Additional file 1 — Digital whiteboard prototype. [file 1472-6947-14-27-S1.zip › gfx/clock/2-1.png]

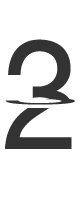

Supplement: Additional file 1 — Digital whiteboard prototype. [file 1472-6947-14-27-S1.zip › gfx/clock/2-2.png]

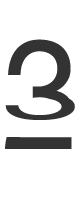

Supplement: Additional file 1 — Digital whiteboard prototype. [file 1472-6947-14-27-S1.zip › gfx/clock/2-3.png]

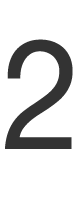

Supplement: Additional file 1 — Digital whiteboard prototype. [file 1472-6947-14-27-S1.zip › gfx/clock/2.png]

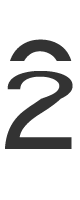

Supplement: Additional file 1 — Digital whiteboard prototype. [file 1472-6947-14-27-S1.zip › gfx/clock/21-1.png]

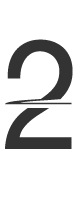

Supplement: Additional file 1 — Digital whiteboard prototype. [file 1472-6947-14-27-S1.zip › gfx/clock/21-2.png]

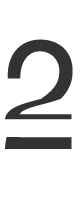

Supplement: Additional file 1 — Digital whiteboard prototype. [file 1472-6947-14-27-S1.zip › gfx/clock/21-3.png]

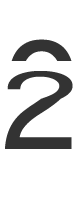

Supplement: Additional file 1 — Digital whiteboard prototype. [file 1472-6947-14-27-S1.zip › gfx/clock/211-1.png]

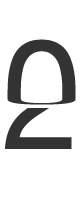

Supplement: Additional file 1 — Digital whiteboard prototype. [file 1472-6947-14-27-S1.zip › gfx/clock/211-2.png]

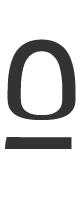

Supplement: Additional file 1 — Digital whiteboard prototype. [file 1472-6947-14-27-S1.zip › gfx/clock/211-3.png]

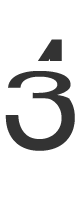

Supplement: Additional file 1 — Digital whiteboard prototype. [file 1472-6947-14-27-S1.zip › gfx/clock/3-1.png]

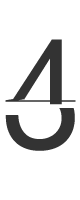

Supplement: Additional file 1 — Digital whiteboard prototype. [file 1472-6947-14-27-S1.zip › gfx/clock/3-2.png]

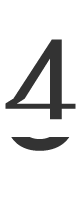

Supplement: Additional file 1 — Digital whiteboard prototype. [file 1472-6947-14-27-S1.zip › gfx/clock/3-3.png]

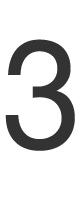

Supplement: Additional file 1 — Digital whiteboard prototype. [file 1472-6947-14-27-S1.zip › gfx/clock/3.png]

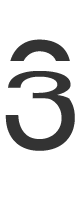

Supplement: Additional file 1 — Digital whiteboard prototype. [file 1472-6947-14-27-S1.zip › gfx/clock/31-1.png]

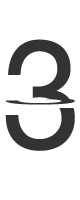

Supplement: Additional file 1 — Digital whiteboard prototype. [file 1472-6947-14-27-S1.zip › gfx/clock/31-2.png]

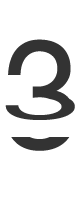

Supplement: Additional file 1 — Digital whiteboard prototype. [file 1472-6947-14-27-S1.zip › gfx/clock/31-3.png]

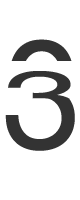

Supplement: Additional file 1 — Digital whiteboard prototype. [file 1472-6947-14-27-S1.zip › gfx/clock/311-1.png]

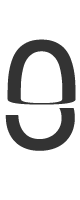

Supplement: Additional file 1 — Digital whiteboard prototype. [file 1472-6947-14-27-S1.zip › gfx/clock/311-2.png]

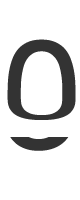

Supplement: Additional file 1 — Digital whiteboard prototype. [file 1472-6947-14-27-S1.zip › gfx/clock/311-3.png]

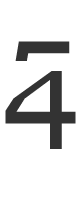

Supplement: Additional file 1 — Digital whiteboard prototype. [file 1472-6947-14-27-S1.zip › gfx/clock/4-1.png]

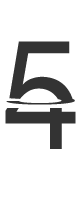

Supplement: Additional file 1 — Digital whiteboard prototype. [file 1472-6947-14-27-S1.zip › gfx/clock/4-2.png]

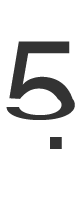

Supplement: Additional file 1 — Digital whiteboard prototype. [file 1472-6947-14-27-S1.zip › gfx/clock/4-3.png]

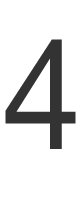

Supplement: Additional file 1 — Digital whiteboard prototype. [file 1472-6947-14-27-S1.zip › gfx/clock/4.png]

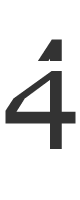

Supplement: Additional file 1 — Digital whiteboard prototype. [file 1472-6947-14-27-S1.zip › gfx/clock/41-1.png]

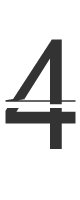

Supplement: Additional file 1 — Digital whiteboard prototype. [file 1472-6947-14-27-S1.zip › gfx/clock/41-2.png]

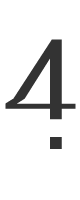

Supplement: Additional file 1 — Digital whiteboard prototype. [file 1472-6947-14-27-S1.zip › gfx/clock/41-3.png]

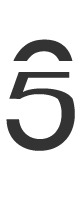

Supplement: Additional file 1 — Digital whiteboard prototype. [file 1472-6947-14-27-S1.zip › gfx/clock/5-1.png]

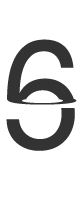

Supplement: Additional file 1 — Digital whiteboard prototype. [file 1472-6947-14-27-S1.zip › gfx/clock/5-2.png]

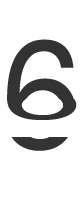

Supplement: Additional file 1 — Digital whiteboard prototype. [file 1472-6947-14-27-S1.zip › gfx/clock/5-3.png]

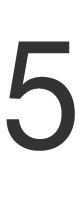

Supplement: Additional file 1 — Digital whiteboard prototype. [file 1472-6947-14-27-S1.zip › gfx/clock/5.png]

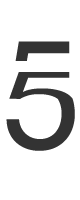

Supplement: Additional file 1 — Digital whiteboard prototype. [file 1472-6947-14-27-S1.zip › gfx/clock/51-1.png]

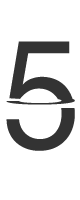

Supplement: Additional file 1 — Digital whiteboard prototype. [file 1472-6947-14-27-S1.zip › gfx/clock/51-2.png]

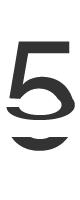

Supplement: Additional file 1 — Digital whiteboard prototype. [file 1472-6947-14-27-S1.zip › gfx/clock/51-3.png]

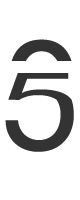

Supplement: Additional file 1 — Digital whiteboard prototype. [file 1472-6947-14-27-S1.zip › gfx/clock/511-1.png]

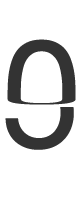

Supplement: Additional file 1 — Digital whiteboard prototype. [file 1472-6947-14-27-S1.zip › gfx/clock/511-2.png]

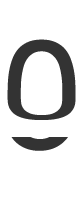

Supplement: Additional file 1 — Digital whiteboard prototype. [file 1472-6947-14-27-S1.zip › gfx/clock/511-3.png]

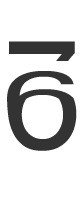

Supplement: Additional file 1 — Digital whiteboard prototype. [file 1472-6947-14-27-S1.zip › gfx/clock/6-1.png]

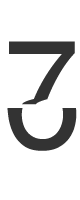

Supplement: Additional file 1 — Digital whiteboard prototype. [file 1472-6947-14-27-S1.zip › gfx/clock/6-2.png]

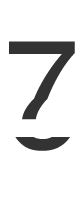

Supplement: Additional file 1 — Digital whiteboard prototype. [file 1472-6947-14-27-S1.zip › gfx/clock/6-3.png]

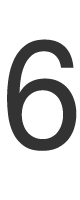

Supplement: Additional file 1 — Digital whiteboard prototype. [file 1472-6947-14-27-S1.zip › gfx/clock/6.png]

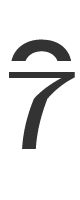

Supplement: Additional file 1 — Digital whiteboard prototype. [file 1472-6947-14-27-S1.zip › gfx/clock/7-1.png]

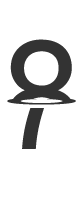

Supplement: Additional file 1 — Digital whiteboard prototype. [file 1472-6947-14-27-S1.zip › gfx/clock/7-2.png]

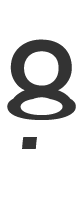

Supplement: Additional file 1 — Digital whiteboard prototype. [file 1472-6947-14-27-S1.zip › gfx/clock/7-3.png]

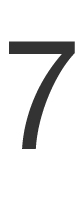

Supplement: Additional file 1 — Digital whiteboard prototype. [file 1472-6947-14-27-S1.zip › gfx/clock/7.png]

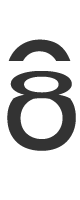

Supplement: Additional file 1 — Digital whiteboard prototype. [file 1472-6947-14-27-S1.zip › gfx/clock/8-1.png]

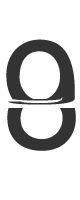

Supplement: Additional file 1 — Digital whiteboard prototype. [file 1472-6947-14-27-S1.zip › gfx/clock/8-2.png]

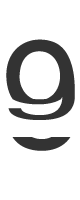

Supplement: Additional file 1 — Digital whiteboard prototype. [file 1472-6947-14-27-S1.zip › gfx/clock/8-3.png]

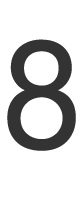

Supplement: Additional file 1 — Digital whiteboard prototype. [file 1472-6947-14-27-S1.zip › gfx/clock/8.png]

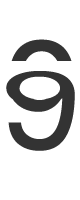

Supplement: Additional file 1 — Digital whiteboard prototype. [file 1472-6947-14-27-S1.zip › gfx/clock/9-1.png]

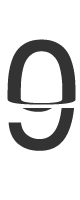

Supplement: Additional file 1 — Digital whiteboard prototype. [file 1472-6947-14-27-S1.zip › gfx/clock/9-2.png]

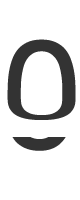

Supplement: Additional file 1 — Digital whiteboard prototype. [file 1472-6947-14-27-S1.zip › gfx/clock/9-3.png]

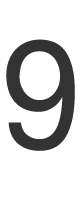

Supplement: Additional file 1 — Digital whiteboard prototype. [file 1472-6947-14-27-S1.zip › gfx/clock/9.png]

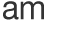

Supplement: Additional file 1 — Digital whiteboard prototype. [file 1472-6947-14-27-S1.zip › gfx/clock/am.png]

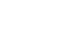

Supplement: Additional file 1 — Digital whiteboard prototype. [file 1472-6947-14-27-S1.zip › gfx/clock/apm.png]

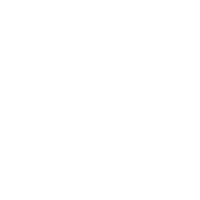

Supplement: Additional file 1 — Digital whiteboard prototype. [file 1472-6947-14-27-S1.zip › gfx/clock/clockbg-blank.png]

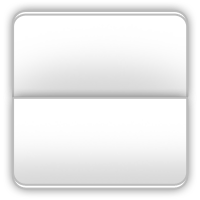

Supplement: Additional file 1 — Digital whiteboard prototype. [file 1472-6947-14-27-S1.zip › gfx/clock/clockbg1.png]

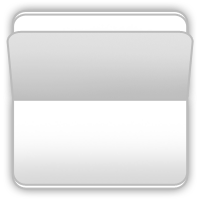

Supplement: Additional file 1 — Digital whiteboard prototype. [file 1472-6947-14-27-S1.zip › gfx/clock/clockbg2.png]

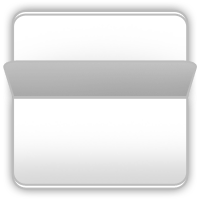

Supplement: Additional file 1 — Digital whiteboard prototype. [file 1472-6947-14-27-S1.zip › gfx/clock/clockbg3.png]

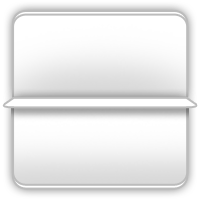

Supplement: Additional file 1 — Digital whiteboard prototype. [file 1472-6947-14-27-S1.zip › gfx/clock/clockbg4.png]

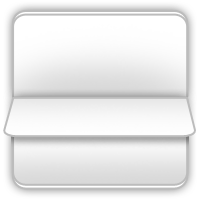

Supplement: Additional file 1 — Digital whiteboard prototype. [file 1472-6947-14-27-S1.zip › gfx/clock/clockbg5.png]

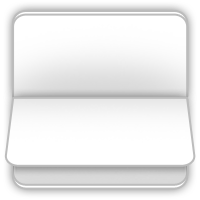

Supplement: Additional file 1 — Digital whiteboard prototype. [file 1472-6947-14-27-S1.zip › gfx/clock/clockbg6.png]

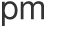

Supplement: Additional file 1 — Digital whiteboard prototype. [file 1472-6947-14-27-S1.zip › gfx/clock/pm.png]

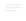

Supplement: Additional file 1 — Digital whiteboard prototype. [file 1472-6947-14-27-S1.zip › gfx/comment.png]

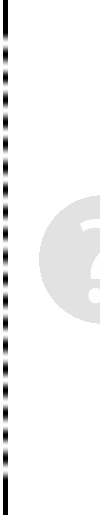

Supplement: Additional file 1 — Digital whiteboard prototype. [file 1472-6947-14-27-S1.zip › gfx/event_gate.png]

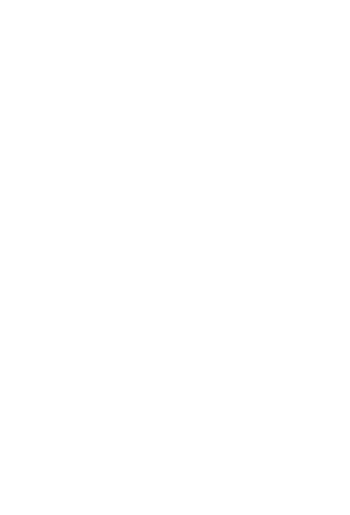

Supplement: Additional file 1 — Digital whiteboard prototype. [file 1472-6947-14-27-S1.zip › gfx/gradient-large.png]

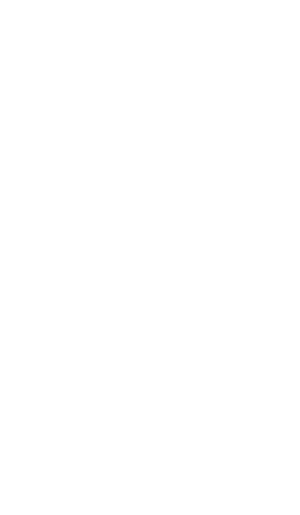

Supplement: Additional file 1 — Digital whiteboard prototype. [file 1472-6947-14-27-S1.zip › gfx/gradient.png]

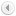

Supplement: Additional file 1 — Digital whiteboard prototype. [file 1472-6947-14-27-S1.zip › gfx/icon_left.png]

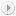

Supplement: Additional file 1 — Digital whiteboard prototype. [file 1472-6947-14-27-S1.zip › gfx/icon_right.png]

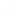

Supplement: Additional file 1 — Digital whiteboard prototype. [file 1472-6947-14-27-S1.zip › gfx/journal.png]

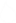

Supplement: Additional file 1 — Digital whiteboard prototype. [file 1472-6947-14-27-S1.zip › gfx/lab.png]

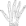

Supplement: Additional file 1 — Digital whiteboard prototype. [file 1472-6947-14-27-S1.zip › gfx/radiology.png]

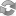

Supplement: Additional file 1 — Digital whiteboard prototype. [file 1472-6947-14-27-S1.zip › gfx/refresh_01.png]

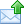

Supplement: Additional file 1 — Digital whiteboard prototype. [file 1472-6947-14-27-S1.zip › gfx/send.png]

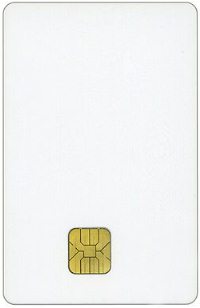

Supplement: Additional file 1 — Digital whiteboard prototype. [file 1472-6947-14-27-S1.zip › gfx/smartcard.jpg]

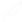

Supplement: Additional file 1 — Digital whiteboard prototype. [file 1472-6947-14-27-S1.zip › gfx/surgery.png]

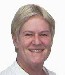

Supplement: Additional file 1 — Digital whiteboard prototype. [file 1472-6947-14-27-S1.zip › gfx/user.jpg]

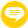

Supplement: Additional file 1 — Digital whiteboard prototype. [file 1472-6947-14-27-S1.zip › gfx/_comment.png]

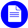

Supplement: Additional file 1 — Digital whiteboard prototype. [file 1472-6947-14-27-S1.zip › gfx/_journal.png]

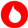

Supplement: Additional file 1 — Digital whiteboard prototype. [file 1472-6947-14-27-S1.zip › gfx/_lab.png]
